# Stable isotope and dental caries data reveal abrupt changes in subsistence economy in ancient China in response to global climate change

**DOI:** 10.1371/journal.pone.0218943

**Published:** 2019-07-22

**Authors:** Christina Cheung, Hua Zhang, Joseph C. Hepburn, Dongya Y. Yang, Michael P. Richards

**Affiliations:** 1 Department of Archaeology, Simon Fraser University, Burnaby, B.C., Canada; 2 Department of Archaeology and Museology, College of History, Nankai University, Jinnan District, Tianjin City, People’s Republic of China; Institute for Anthropological Research, CROATIA

## Abstract

Prior to the introduction of wheat and barley from Central Asia during the Neolithic period, northern Chinese agricultural groups subsisted heavily on millet. Despite being the focus of many decades of intensive interest and research, the exact route(s), date(s), and mechanisms of the spread and adoption of wheat and barley into the existing well-established millet-based diet in northern China are still debated. As the majority of the important introduced crops are C_3_ plants, while the indigenous millet is C_4_, archaeologists can effectively identify the consumption of any introduced crops using stable carbon isotope analysis. Here we examine published stable isotope and dental caries data of human skeletal remains from 77 archaeological sites across northern and northwestern China. These sites date between 9000 to 1750 BP, encompassing the period from the beginning of agriculture to wheat’s emergence as a staple crop in northern China. The aim of this study is to evaluate the implications of the spread and adoption of these crops in ancient China. Detailed analysis of human bone collagen *δ*^13^C values reveals an almost concurrent shift from a C_4_-based to a mixed C_3_/ C_4_– based subsistence economy across all regions at around 4500–4000 BP. This coincided with a global climatic event, Holocene Event 3 at 4200 BP, suggesting that the sudden change in subsistence economy across northern and northwestern China was likely related to climate change. Moreover, the substantially increased prevalence of dental caries from pre–to post–4000 BP indicates an increase in the consumption of cariogenic cereals during the later period. The results from this study have significant implications for understanding how the adoption of a staple crop can be indicative of large-scale environmental and socio-political changes in a region.

## 1. Introduction

Beginning in the Neolithic period, China has traditionally been divided by an invisible “crop line.” The phrase “south rice north millet” 南稻北粟 has often been used to describe the phenomenon whereby communities north of the Qin Mountains and Huaihe River were known to subsist on a millet-based diet, while those located south of these landmarks subsisted primarily on a rice-based diet [1, 2]. This division of agro-cultural zones was shaped first and foremost by the different climatic and geographic conditions of northern and southern China, as well as a combination of contact histories and differing socio-cultural factors [1, 3].

Millet is a C_4_ crop, while rice, wheat, and barley are all C_3_ crops. As plants using the C_3_ and C_4_ photosynthetic pathways are known to have very different carbon isotopic signatures, the dietary differences between the groups in different parts of China are not only culinary in nature, but are also visible isotopically. Thus, stable carbon isotope analysis of human tissues can be an incredibly powerful tool to directly identify, as well as quantify, the amount of C_3_– (e.g. wheat, barley, rice) versus C_4_– (e.g. millet) based food in these different groups’ diets [4–7].

Moreover, proxies of ancient dental health, particularly the prevalence of dental caries, are a powerful tool for archaeologists to monitor changes in carbohydrate intake of different prehistoric populations [8–11]. The data generated do not simply reflect changes in diets, such as the increasing proportion of certain grains in diets; they can also reflect fundamental changes in a group’s subsistence economic system [12–14]. Here we present a study that utilizes both stable isotope analysis and an examination of dental caries to shed light on the lifestyle, diet, nutrition, and subsistence economies of past populations. Although each method has its own advantages, several previous studies have demonstrated the complementary power of stable isotopic and dental health data when used together [15–17]. Such approaches can further enhance the resolution of the reconstruction, thus permitting a better understanding of the general trend of subsistence economy through time. We have collated stable isotopic and dental caries data from a total of 77 sites, spanning over a period of 7250 years and traversing eight modern provinces. From the data, we report diachronic trends in terms of isotopic measurements as well as dental health in response to the incorporation of C_3_ crops into northern and northwestern Chinese diets, and discuss these patterns within their larger socio-political and geographic contexts.

## 2. Archaeological context

### 2.1 Cereal crops in early northern and northwestern China

By the Bronze Age (ca. 4000–2000 BP), common cereal cultivars in northern and northwestern China included millet (both *Panicum miliaceum* and *Setaria italica* varieties), wheat (*Triticum aestivum*), barley (*Hordeum vulgare*), and rice (*Oryza sativa*) [18–25]. Other cultivars such as acorn (*Quercus* spp.), soybeans (*Glycine soja*), and various tubers, were also prevalent, albeit in much smaller quantities [20, 23, 26, 27]. Among the four most important cereal cultivars, millet is the only one native to northern China, and is generally considered to be one of the first domesticated crops in China [28–30]. During the early Neolithic period (10,000–8000 BP), millet agriculture spread across northern China very quickly and by the late Neolithic period (5000–4000 BP), was the predominant subsistence economy in most parts of northern as well as northwestern China [27, 30–32]. Rice, wheat, and barley were introduced into the region variously during the late Neolithic period.

Among the three imported crops, rice generally requires a wetter and warmer environment to grow, and therefore has been traditionally considered a southern Chinese crop (southern China is commonly defined as south of Huaihe River) [1, 33, 34]. However, sporadic palaeobotanical finds in northern China dating to as early as the late Neolithic period suggest there was limited rice production in selected regions in northern China [22, 35, 36]. Nevertheless, due to climatic and environmental constraints, large-scale rice agriculture was never widespread in northern and northwestern China during prehistoric and early historic times.

Wheat and barley were introduced to northern China from Central Asia during the late Neolithic to early Bronze Age [19, 20, 37–39]. In the literature, wheat and barley are often discussed together as a “package”. As they originated from the same region and have very similar growing requirements, many archaeologists have assumed they travelled together [39]. However, some recent studies have suggested that wheat and barley may have travelled to northwestern and northern China at different times, and were received by communities differently. Archaeological evidence from several sites in the Xinjiang and Gansu regions showed that wheat started to appear in northwestern China as early as 5000 BP, while barley likely arrived a few hundred years later [18, 20, 21, 40]. Furthermore, palaeobotanical evidence revealed that wheat was recovered in significantly larger quantities than barley in most northern and northwestern Chinese sites [41], suggesting wheat was being consumed in greater proportions. While it is certain from historical records that by the Tang Dynasty (618–907 AD), the consumption of wheat had largely surpassed millet and become the staple cereal in northern China [42, 43], the rate and the nature of how and why this shift in subsistence economy occurred is still under debate [6, 19, 44–46].

Even though it is not possible to distinguish between the consumption of the three imported crops: wheat, barley, and rice in ancient populations based on stable isotopic and dental health data alone, both palaeobotanical and historical records suggest that out of these three cereals, wheat was most widely adopted in early China. Therefore, much of the argument in this study will be based on the assumption that a large proportion of C_3_ signal in populations post-5000 BC was caused by the consumption of wheat.

### 2.2 Wheat and millet

Millet and wheat have different optimal growing requirements. Millet is often grown in semiarid regions due to its ability to grow under high heat and drought conditions [47, 48]. Wheat, specifically the strains known as winter wheat, grows better in the cooler season, and requires relatively little labour in its cultivation [49]. These two crops also have very different growth cycles. Millet is often sown during the early summer months, and can be harvested in 75–100 days after seeding [50]. Winter wheat is usually sown at the beginning of the winter, and can take up to eight months to fully mature [51]. Due to their alternating optimal sowing time, in modern farming systems, millet is often incorporated into a crop rotation system with winter wheat [52, 53].

In terms of nutrition, millet and wheat have comparable macro- and micro- nutrient profiles [54, 55]. However, unlike millet, which can be consumed with minimal processing, whole-wheat needs to be processed prior consumption to improve digestibility as well as palatability (e.g. milled, rolled, grind, cracked, fermented, and etc.). Nonetheless, winter wheat consistently gives a higher yield of grain per acre than millet [56], and is culinarily versatile. In contrast to millet, wheat grains contain a special composite of proteins called gluten [54, 57]. By altering the consistency of wheat flour, and hence the strength and elasticity of gluten, pastries of a variety of textures and forms can be created. Although isolated cases of noodles and various pastries have been discovered in late Neolithic and early Bronze Age contexts in northwestern China [58–60], there is little evidence these foods were widespread in northern China during that time. In fact, the only mention of wheat consumption in early historical literary sources is as wheat gruel (麥飯) [61]. The term “processed wheat food” (*mianshi* 麵食) is not mentioned until the late Warring States period and Western Han Dynasty [62]. While other cereals, such as millet and rice, were also consumed as gruel, gruel made with unrefined wheat is tougher to digest, and therefore was generally considered unpalatable [63]. As a result, wheat gruel was frequently associated with people of lower socioeconomic status [62, 63]. For example, a passage in book two of the Han book *Jijiupian* (急就篇) stated that wheat gruel and bean soup were considered food for the barbarians and farmers (麥飯豆羹皆野人農夫之食耳) [64]. This belief was only overturned in the Tang Dynasty (618–907 AD), when “foreign food” (*hushi* 胡食) from Central Asia—including various highly processed wheat products—became fashionable in northern China [62, 63]. Since then, wheat rapidly became the major staple crop in northern China [42, 43].

## 3. Materials and methods

### 3.1 Temporal and spatial span of study

The time period under scrutiny in this study spans from the mid-Neolithic period (c. 9000 BP), to after the collapse of the Eastern Han Dynasty (220 AD) (Table 1). This timeline of our dataset is constrained firstly by the paucity of data (both isotopic and osteological) from sites prior to 9000 BP. Secondly, by the profoundly fragmentary political climate in Northern China after the collapse of the Eastern Han Dynasty. This chaotic period, known as the Sixteen Kingdoms Period (304–439 AD) was a marked departure from the relative stability of the Han Dynasties, in which the formerly unified northern China was split into 16 different states ruled by several different ethnic groups. The political heterogeneity in this period effectively eliminates the ability to sensibly constrain and account for factors when looking at changes in dietary practices in this region.

**Table 1:** The approximate chronology of different cultural groups across northern China.

Phase	Year (BP)	Major Cultural Period
1	9000–8500	**Early Neolithic Period**: Jiahu Culture, Houli Culture, Laoguantai Culture
2	8500–8000	
3	8000–7500	
4	7500–7000	
5	7000–6500	**Middle Neolithic Period**: Yangshao Culture, Banpo Culture, Shijia Culture, Miaodigou Culture, Dawenkou Culture
6	6500–6000	
7	6000–5500	
8	5500–5000	
9	5000–4500	**Late Neolithic period**: Qujialing, Dawenkou, Shijiahe, Longshan, Kexingzhuang, Banshan, Machang, Qijia, Zongri, Majiayao
10	4500–4000	
11	4000–3500	Proto-Shang/ Xia Dynasty, Qijia, Siba, Siwa, Machang
12	3500–3000	Shang Dynasty, Predynastic Zhou, Siwa, Siba, Qijia, Xindian, Kayue
13	3000–2500	Western Zhou Dynasty–Spring-Autumn period (early E. Zhou), Shanrong Culture
14	2500–2000	Warring States period (late E. Zhou), Qin Dynasty, Western Han Dynasty
15	2000–1750	Eastern Han Dynasty

It is a common practice for Chinese archaeologists to discuss all prehistoric periods in a BP framework, and all historical (late Shang Dynasty and after) periods in a BC/AD framework. Since this study engages with archaeological populations dating from early prehistory to historical dynasties, this traditional approach would result in a confusing mixture of time scales. Furthermore, very few of the samples discussed in this study were directly dated. For the majority of the sites, time periods were assigned based on typological analysis of associated artifacts. While we are in no position to evaluate the accuracy of this dating method, in the cases of historical sites, we feel that providing an absolute date range (e.g. 475–221 BC for the Warring States period) could be misleading. Thus, in order to ensure the chronology of sites are quoted consistently and broadly accurate, the period in study is divided into 15 phases, where each phase represents a 500 year-period, while the last phase only covers about 220 years (see Table 1).

**Fig 1:**
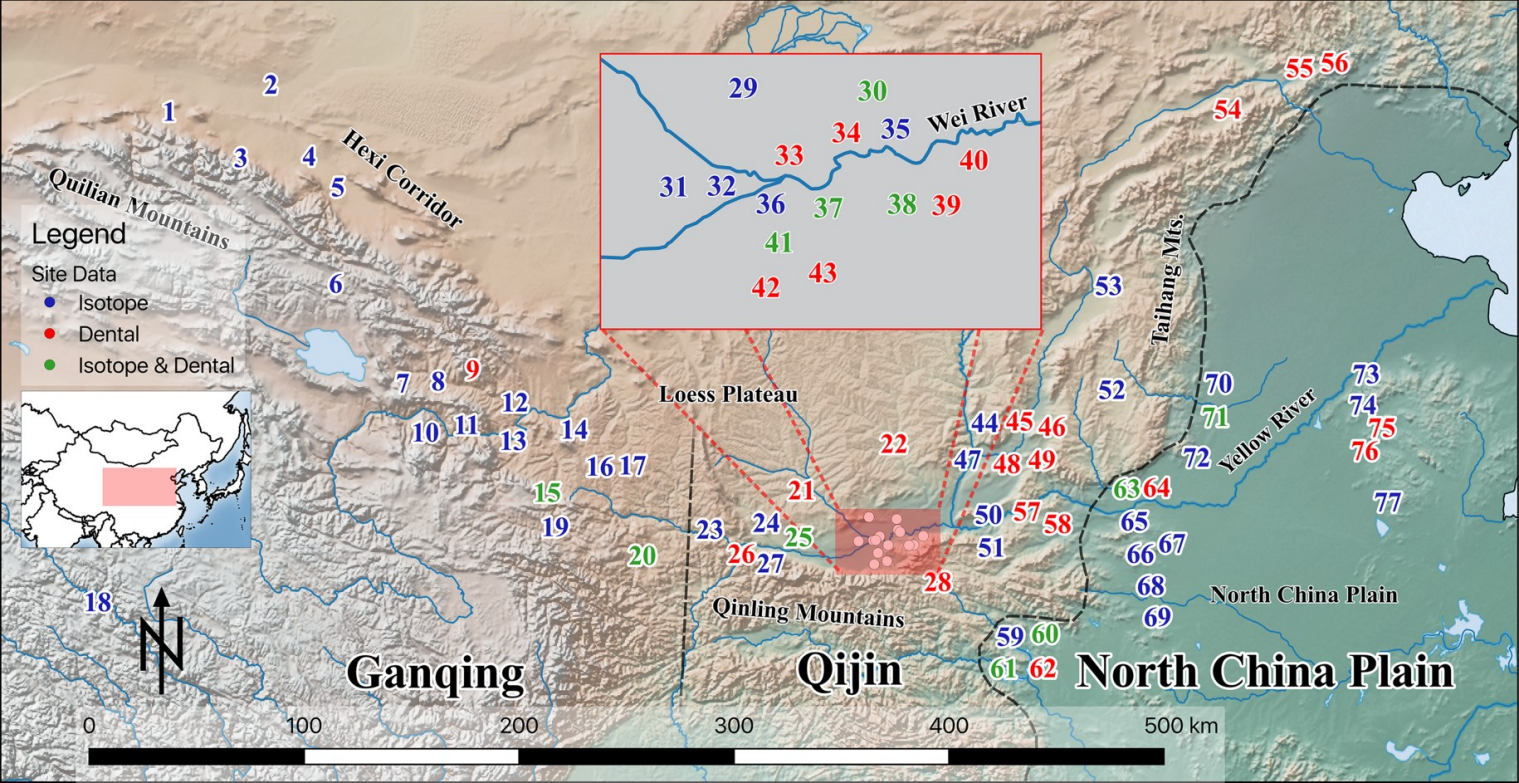
A map showing all sites analysed in this study and the major geological features of northern and northwestern China. A key to site IDs is provided in the Table 2. The map was created using QGIS Version 2.12.0 for OSX (www.qgis.org). All maps used are in the public domain (CC0), sourced from Natural Earth (www.naturalearthdata.com).

As the geological and climatic conditions in the study area are so dramatically diverse, it is expected that the subsistence strategies adopted by populations in different regions would be vastly different. In order to effectively control for environmental variables, we have divided the study area into three broad geographical zones: the North China Plain (NCP, 33°-40°N, 110°-117°E), the Qinjin region (QJ, 33°-36°N, 106°-112°E), and the Ganqing region (GQ, 33°-40°N, 96°°-108°E). NCP refers to the part of northern China located to east of the Taihang mountain range, includes parts of the modern-day Henan, Hebei, northern Hubei, and Shandong provinces. QJ refers to the part of Northern China centring around the middle reaches of the Yellow River and west of the Taihang mountain range, including the modern-day Shaanxi, Shanxi, as well as eastern Henan and Hebei provinces. GQ refers to the mountainous area between the Loess and Tibetan plateaus, includes both the modern provinces of Gansu and Qinghai. It is important to note that this approach departs from traditional conventions in historical and archaeological literature, where the two eastern regions, NCP and QJ, are often jointly refer to as northern China, and GQ is traditionally considered as part of northwest China. Topographically, all sites located in NCP are located on relatively flat land, with elevations below 200m a.s.l. Sites in QJ are located >300m a.s.l. and <1000m a.s.l. (with Neiyangyuan being an exception). Sites in GQ are located in areas with elevation >1000m a.s.l. The elevations of sites were obtained from mapcoordinates.net (https://www.mapcoordinates.net/en), and are only provided to help organize sites into the most appropriate geographical zone. As very few site reports provided coordinates, most of the site locations are estimated from textual descriptions. Consequently, most site coordinates and elevations should only be treated as a general reference. All in all, we hope to control for as much geographic variations as possible, so that the differences in our collected data can be isolated and attributed to differences in subsistence economies. All sites involved are listed in Table 2 and plotted in Fig 1.

**Table 2:** Names, references (in brackets), cultural phases, coordinates (latitudes and longitudes), and elevations of all sites included in the database. For the time period of each cultural phase please refer to Table 1. Site ID corresponds to number shown on Fig 1. NCP refers to North China Plain, QJ refers to the Qinjin region, GQ refers to the Ganqing region. For more detailed description of each region please refer to section 3.

Site		Location	Cultural phase	Region	Site ID	Latitude	Longitude	Elevation (m a.s.l)	Dental Caries data	Stable isotope data
Huoshaogou [65]	火燒溝	Gansu	Siba Culture	GQ	1	39.960279	97.655051	1761		*
Huoshiliang [66]	火石梁	Gansu	Siba Culture	GQ	2	40.26	99.305	1195		*
Ganguya [65]	乾骨崖	Gansu	Siba Culture	GQ	3	39.382711	98.856553	1827		*
Wuba [65]	五壩	Gansu	Banshan–Machang Cultures	GQ	4	39.380785	99.890372	1360		*
Xichengyi [67]	西城驛	Gansu	Machang–Siba Cultures	GQ	5	39.014436	100.365415	1460		*
Mozuizi [65, 68]	磨嘴子	Gansu	Machang Culture	GQ	6	37.786062	100.365415	4155		*
Hupo [45]	護坡	Gansu	Banshan–Machang Cultures	GQ	7	36.4	102	2512		*
Sanheyi [45]	三合乙	Gansu	Qijia Culture	GQ	8	36.4	102	2512		*
Liuwan [69]	柳灣	Qinghai	Majiayao Culture	GQ	9	36.445537	102.561661	1922	*	
Wenbuju [68]	文卜具	Qinghai	Majiayao Culture	GQ	10	36	102	2000		*
Lajigai [45]	拉吉蓋	Gansu	Kayue Culture	GQ	11	36	102.3	2382		*
Xiahaishi [5, 70]	下海石	Gansu	Machang Culture	GQ	12	36.344608	102.856376	1771		*
Lianhuatai [5]	蓮花台	Gansu	Xindian Culture	GQ	13	35.769601	103.165769	1757		*
Qijiaping [71]	齊家坪	Gansu	Qijia Culture	GQ	14	35.887345	104.062574	2037		*
Mogou [45, 65, 72]	磨溝	Gansu	Qijia–Siwa Cultures	GQ	15	34.977773	103.780975	2348	*	*
Buziping [70]	堡子坪	Gansu	Qijia Culture	GQ	16	35.4	104.5	2298		*
Buzishan [70]	堡子山	Gansu	Qijia Culture	GQ	17	35.4	104.5	2298		*
Zongri [73]	宗日	Qinghai	Zongri Culture	GQ	18	33.552134	96.380682	4242		*
Zhanqi [5, 65]	占旗	Gansu	Siwa Culture	GQ	19	34.714335	103.844992	2263		*
Xishan [74, 75]	西山	Gansu	Western Zhou to Warring States	GQ	20	34.192296	105.183033	1414	*	*
Nianzipo [76]	碾子坡	Shaanxi	Predynastic Zhou to early Western Zhou	WCP	21	35.125871	107.902396	918	*	
Zhaitouhe [77]	寨頭河	Shaanxi	Warring States period	WCP	22	35.677378	109.330648	829	*	
Jianhe [75]	建河	Shaanxi	Warring States period	WCP	23	34.515439	106.364578	964		*
Sunjianantou [78]	孫家南頭	Shaanxi	Eastern Zhou	WCP	24	34.472717	107.24411	686		*
Zhouyuan [5, 75, 76]	周原	Shaanxi	Predynastic Zhou to early Western Zhou	WCP	25	34.486595	107.602417	774	*	*
Beishouling [69]	北首嶺	Shaanxi	Yangshao Culture	WCP	26	34.384346	107.15891	600	*	
Shigushan [5]	石鼓山	Shaanxi	Predynastic Zhou to early Western Zhou	WCP	27	34.343362	107.190987	607		*
Donglongshan [76]	東龍山	Shaanxi	Xia–Shang Dynasties	WCP	28	33.845756	109.969282	708	*	
Guandao [79]	官道	Shaanxi	Mid-Western Han to Eastern Han	WCP	29	34.752401	108.90653	629		*
Shijia [80, 81]	史家	Shaanxi	Shijia Culture	WCP	30	34.725018	109.357346	384	*	*
Jichang [79]	機場	Shaanxi	Eastern Han	WCP	31	34.429958	108.738685	488		*
Guangming [79]	光明	Shaanxi	Western Han	WCP	32	34.440213	108.976327	405		*
Podi [82]	坡底	Shaanxi	Qin Dynasty	WCP	33	34.493787	109.0803	377	*	
Kangjia [80]	康家	Shaanxi	Longshan Culture	WCP	34	34.581685	109.378182	360	*	
Baijia [83]	白家	Shaanxi	Laoguantai Culture	WCP	35	34.55209	109.4107	350		*
Dongying [84]	東營	Shaanxi	Kexingzhuang II	WCP	36	34.44333	109.0153	374		*
Jiangzhai [80, 81, 85]	姜寨	Shaanxi	Banpo and Shijia Cultures	WCP	37	34.377858	109.218143	446	*	*
Beiliu [80, 86]	北劉	Shaanxi	Laoguantai and Miaodigou cultures	QJ	38	34.374866	109.555338	583	*	*
Dongyang [76]	東陽	Shaanxi	Western Zhou to Han Dynasty	WCP	39	34.380953	109.633293	605	*	
Baoji Huaxian [87]	寶雞華縣	Shaanxi	Early Yangshao Culture	WCP	40	34.499118	109.78697	409	*	
Banpo [81, 88]	半坡	Shaanxi	Banpo Culture	WCP	41	34.2729	109.053402	421	*	*
Shaolingyuan [89]	少陵原	Shaanxi	Western Zhou	WCP	42	34.120894	108.992837	489	*	
Qujia Wanlijia [90]	屈家 灣李家	Shaanxi	Warring States to Qin Dynasty	WCP	43	34.162486	109.199544	693	*	
Neiyangyuan [91]	內陽垣	Shanxi	Xia and Spring-Autumn	WCP	44	35.98988	110.785445	1079		*
Taosi [76]	陶寺	Shanxi	Longshan Culture	WCP	45	35.87855	111.496386	593	*	
Qucun [76]	曲村	Shanxi	Western Zhou	WCP	46	35.731214	111.554966	496	*	
Liangdaicun [75, 92]	梁帶村	Shaanxi	Western Zhou to Spring-Autumn	WCP	47	35.507316	110.502839	366		*
Shangma [76]	上馬	Shanxi	Spring-Autumn	WCP	48	35.590366	111.349213	414	*	
Qiaocun [76]	喬村	Shanxi	Eastern Zhou	WCP	49	35.615436	111.410459	432	*	
Qiangliang Temple [93]	清涼寺	Shanxi	Miaodigou and Longshan cultures	WCP	50	34.76158	110.894048	532		*
Xipo [94]	西坡	Henan	Yangshao Culture	WCP	51	34.35444	110.846353	871		*
Tunliu Yuwu [95]	屯留余吾	Shanxi	Warring States and the Han Dynasties	WCP	52	36.376475	112.843458	964		*
Neidan [96]	聶店	Shanxi	Xia Dynasty	WCP	53	37.751272	112.741538	855		*
Sanguan [69]	三關	Hebei	Lower Xiajiadian Culture	WCP	54	39.925189	114.78789	924	*	
Xitun [97]	西屯	Hebei	Han Dynasty	WCP	55	40.453251	115.941883	484	*	
Yanqing [76]	延慶	Hebei	Shanrong Culture	WCP	56	40.456744	115.974986	483	*	
Miaodigou [69]	廟底溝	Henan	Miaodigou Culture	ECP	57	34.769843	111.167221	357	*	
Duzhong [98]	篤忠	Henan	Late Yangshao Culture	ECP	58	34.667016	111.866047	477	*	
Shenmingpu [25]	申明鋪	Henan	Warring States and the Han Dynasties	ECP	59	33.002771	111.303279	166		*
Gouwan [99, 100]	溝灣	Henan	Yangshao and Qujialing Cultures	ECP	60	33.078699	111.47917	176	*	*
Qinglongquan [101–103]	青龍泉	Hubei	Qujialing and Shijiahe Cultures, Eastern Zhou	ECP	61	32.83979	110.851701	189	*	*
Xiawanggang [104]	下王崗	Henan	Early Yangshao Culture	ECP	62	33.016686	111.370493	198	*	
Chenjiagou [105, 106]	陳家溝	Henan	Western and Eastern Zhou	ECP	63	34.939501	113.149566	103	*	*
Guangwu [107]	廣武	Henan	Late Yangshao Culture	ECP	64	34.894468	113.445476	116	*	
Xuecun [106]	薛村	Henan	Western and Eastern Han	ECP	65	34.865228	113.238266	141		*
Wadian [108]	瓦店	Henan	Longshan Culture	ECP	66	34.18744	113.4049	130		*
Xinzheng City [106]	新鄭市	Henan	Western and Eastern Zhou	ECP	67	34.396609	113.753075	107		*
Yangdi [106]	陽翟	Henan	Eastern Zhou	ECP	68	33.866937	113.446075	85		*
Jiahu [109]	賈湖	Henan	Jiahu Culture	ECP	69	33.612622	113.667383	70		*
Nancheng [110]	南城	Hebei	Proto-Shang	ECP	70	36.50347	114.375754	81		*
Yinxu [5, 111–114]	殷墟	Henan	Shang Dynasty	ECP	71	36.13944	114.3031	82	*	*
Liuzhuang [115]	劉莊	Henan	Proto-Shang	ECP	72	35.605103	114.132122	98		*
Xiaojingshan [116]	小荊山	Shandong	Houli Culture	ECP	73	36.496515	116.844681	67		*
Liujiazhuang [117]	劉家莊	Shandong	Shang Dynasty	ECP	74	36.421557	116.838755	142		*
Dawenkou [69]	大汶口	Shandong	Dawenkou Culture	ECP	75	35.945517	117.093354	91	*	
Panmiao [118]	潘廟	Shandong	Eastern Han	ECP	76	35.583633	116.811004	48	*	
Xigongqiao [119]	西公橋	Shandong	Dawenkou Culture	ECP	77	34.937363	117.23151	53		*

Data were collected from archaeological sites that span the spectrum of subsistence economies from Neolithic early agriculturalists to dynastic intensified agriculturalists, with varying levels of crop cultivation development. In order to search for relevant datasets, a systematic literature review was conducted using Google Scholar and China National Knowledge Infrastructure (CNKI 中國知網). Only papers or reports published before September 2017 are included. To the best of our knowledge, our sample adequately represents all of the indexed literature on this subject.

Statistical analyses of the results were conducted using R (version 3.0.3). Graphs were created using the package ggplot2 [120]. To help visualize the trends in our stable isotopic data, we used LOESS (locally weighted scatter-plot smoother), a non-parametric regression analytical tool to create smoothed regression lines for the time-series data. These lines were created with the function “geom_smooth” in ggplot2, using the method “loess”, with a confidence interval set at 0.95. Detailed R scripts for all the analyses performed in this study are provided in S1 File–R scripts.

### 3.2 Stable isotope analysis

For an overview of the technique, please refer to S2 File–Supporting Information. As all sites examined in this study are inland/ noncoastal, *δ*^13^C values are primarily used to quantify the relative amount of C_3_– versus C_4_– based food in a group’s diet. Compared to *δ*^13^C values, *δ*^15^N values are more sensitive to environmental variations, especially aridity [121–123]. Considering the vast geographical region covered in this study, the lack of faunal baseline data from many sites means that we are not able to reliably use *δ*^15^N values for the reconstruction of past subsistence practices. As a result, in this study, *δ*^15^N values are only used as a reference point to evaluate the consistency of subsistence practices alongside *δ*^13^C values.

Moreover, bone collagen isotopic compositions primarily reflect the protein portion of diet [124]. As animal products tend to have a higher protein content than plants, it is understood that isotopic signals from animal protein tend to be overrepresented in the bone collagen of omnivores [125]. While it would be ideal to quantify the proportions of the different cereals consumed by each population, it is currently not possible, as that would require having both archaeological plant and animal baseline data from each individual site. Fortunately, several studies have already demonstrated that bone collagen *δ*^13^C and *δ*^15^N values can be used to infer the broader agricultural system of a community: as horticultural by-products are often used as animal feeds, the isotopic composition of domesticates should reflect those of the major cultivars [29, 81, 84, 116, 126]. Thus, this study will mainly use human bone collagen *δ*^13^C values as a proxy to understand the subsistence economy of a group, whether it was largely C_4_– based (i.e. millet-based), C_3_– based (i.e. wheat/barley/rice-based), or a mixture of both.

A total of 1196 (ECP: n = 483; WCP: n = 331; QC: n = 382) human bone collagen C and N measurements from 53 sites (ECP: n = 15; WCP: n = 19; QC: n = 19) are included in this study (Fig 1). While not all papers reported their data along with collagen quality indicators (collagen yield, %C, %N, and atomic C/N ratio), we only include data from papers that reported at least C/N ratios. For those that reported their data with collagen quality indicators, all samples that fall outside of the conventional acceptable ranges have been excluded [127–130]. *δ*^13^C and *δ*^15^N values are reported in ‰ (parts per thousand), calibrated to VPDB and AIR, respectively. While some studies report isotopic measurements at 2 or even 3 decimal places, we standardized all measurements to 1 decimal place for consistency, and to better represent analytical precision. A complete list of all the summary information of the different skeletal series analysed for carbon and nitrogen isotopic compositions in this study is presented in Table 3. The full set of isotopic data analysed in this study is provided in S3 File–Isotope data.

**Table 3:** Means and standard deviations of *δ*^13^C (‰, VPDB) and *δ*^15^N values (‰, AIR) of values from individuals from each phase. Sites with * also have dental pathology data.

	NCP				QJ				GQ			
Year (BP)	Sites	N	*δ*^13^C (‰)	*δ*^15^N (‰)	Sites	N	*δ*^13^C (‰)	*δ*^15^N (‰)	Sites	N	*δ*^13^C (‰)	*δ*^15^N (‰)
9000–8500	Jiahu	5	–19.9 ± 0.7	+8.8 ± 0.8								
8500–8000	Jiahu	4	–20.5 ± 0.2	+9.5 ± 0.9	Beilu	5	–12.2 ± 1.3	+9.0 ± 0.6				
8000–7500	Xiaojingshan	10	–17.8 ± 0.3	+9.0 ± 0.6								
7500–7000					Baijia	1	–14.5	+11.1				
7000–6500	Gouwan	10	–14.5 ± 0.9	+7.2 ± 0.3	Banpo* Jiangzhai I*	8	–10.1 ± 2.1	+8.8 ± 0.6				
6500–6000	Gouwan	20	–14.2 ± 2.5	+8.9 ± 0.9	Jiangzhai II* Shijia*	19	–10.0 ± 0.9	+8.3 ± 0.4				
6000–5500	Gouwan*	6	–15.0 ± 1.7	+8.8 ± 0.5	Beiliu Xipo	34	–9.8 ± 1.2	+9.3 ± 1.0				
5500–5000					Qiangliang Temple	13	–8.4 ± 1.1	+8.1 ± 1.1				
5000–4500	Gouwan Xigongqiao Qinglongquan*	11	–14.8 ± 2.1	+8.8 ± 1.5								
4500–4000	Qinglongquan Wadian	29	–12.9 ± 2.2	+8.6 ± 1.3	Dongying-2 Qiangliang Temple	18	–8.3 ± 1.1	+8.5 ± 1.1	Wuba Mozuizi Wenbuju Hupo Zongri Buziping Buzishan	99	–8.0 ± 1.4	+8.7 ± 1.0
4000–3500	Nancheng Liuzhuang	96	–7.3 ± 1.4	+9.5 ± 0.9	Neiyangyuan Niedian	62	–7.1 ± 0.3	+10.4 ± 0.8	Xichengyi Huoshiliang Sanheyi Xiahaishi Houshaogou Mogou*	138	–12.8 ± 2.8	+9.8 ± 1.8
3500–3000	Liujiazhuang Yinxu*	79	–9.0 ± 1.2	+9.9 ± 1.0	Zhouyuan* Shigushan Liangdaicun	51	–9.2 ± 1.8	+9.5 ± 1.0	Qijiaping Lianhuatai Lajigai Ganguyai Zhanqi Xishan	131	–13.0 ± 3.5	+10.0 ± 1.6
3000–2500	Xinzheng City Qinglongquan Chenjiagou* Yangdi	112	–10.8 ± 2.3	+8.8 ± 1.0	Neiyangyuan Sunjianantou	42	–10.0 ± 1.8	+8.9 ± 1.1				
da2500-2000	Xinzheng City Shenmingpu Xuecun	59	–13.5 ± 2.0	+9.1 ± 1.3	Jianhe Tunliu Yuwu Guangming	29	–9.4 ± 1.0	+9.3 ± 1.1				
2000–1750	Xuecun Shenmingpu	42	–13.9 ± 1.5	+10.3 ± 1.5	Jichang Tunliu Yuwu Guandao	48	–11.5 ± 1.3	+9.2 ± 1.1	Xishan*	14	–11.4 ± 2.3	+9.1 ± 0.8
	**Total**	**483**				**331**				**382**		

### 3.3 Dental caries analysis

To examine the dental health status, and to verify whether and how the prevalence of caries is associated with the introduction of wheat and other cereal crops in ancient Chinese diet, we have taken published dental caries data from 35 sites in northern and northwest China (Fig 1). All identified literature was screened on the basis of the following two key factors: 1) dental caries diagnostic criteria, and 2) prevalence. Given the purpose of this study and the value of including as many sites as possible in the analysis, the frequency (%) compared the number of teeth affected by caries with the number of teeth observed in each population (tooth count). Evaluating the prevalence on each type of tooth was unfortunately not an option at this stage, nor was a comprehensive analysis on the role of age and sex, due to the incomplete reporting conventions in most of the studies. Further discussion on these analytical obstacles, and a tentative investigation on the impact of demographic compositions in dental caries prevalence are provided in S2 File–Supporting Information.

A total of 45,557 teeth from 2934 individuals (number of teeth–NCP: n = 6737; QJ: n = 32,770; QC: n = 6050; number of individuals–NCP: n = 532; QJ: n = 2104; GQ: n = 298) were analysed in this study. Unfortunately, due to the nature of data reporting, it is not possible to organize the dental caries data into the same temporal phases as the stable isotopic data. Based on the inflection points of the stable isotope data, we have chosen to divide our dental data into two phases, representing periods before and after a change in isotopic data. As a result, data are organized in two phases: <4000BP vs. >4000BP.

Due to the wide spread of the data ranges, medians and interquartile ranges (IQR), instead of means and standard deviations, are used to describe the spread of data and minimize any outliers. A complete list of all the skeletal series, sample size, and sources analysed for dental health in this study is presented in Table 4. The full set of dental caries data analysed in this study is provided in S3 File–Dental caries data.

**Table 4:** Summary of dental caries prevalence in samples (teeth affected / total of teeth preserved for examination).

Phase	Year (BP)	Sites	No. of individuals	Teeth Affected / Observed	Percentage (%)
		**NCP Sites**			
>4000BP	7000–6000	Xiawanggang	161	52 / 975	5.3
>4000BP	6000–5000	Gouwan	56	54 / 750	7.2
>4000BP	6500–4500	Qinglongquan	87	128 / 1075	11.9
>4000BP	6100–4500	Dawenkou	17	17 / 510	3.3
>4000BP	5600–5000	Guangwu	15	41 / 210	19.5
<4000BP	3400–3100	Yinxu	118	82 / 1997	4.1
<4000BP	3000–2220	Chenjiagou	61	225 / 982	22.9
<4000BP	2000–1750	Panmiao	17	25 / 238	10.5
** **	** **	**QJ Sites**	** **	** **	
>4000BP	7000–6000	Baoji Huaxian	59	32 / 948	3.4
>4000BP	7000–6000	Jiangzhai	37	11 / 418	2.6
>4000BP	7000–5790	Beishouling	36	28 / 424	6.6
>4000BP	6390	Beiliu	10	4 / 92	4.4
>4000BP	6500–5500	Banpo	73	26 / 913	2.9
>4000BP	6000–5000	Shijia	49	7 / 179	3.9
>4000BP	5500–5000	Duzhong	15	43 / 376	11.4
>4000BP	4900–4800	Miaodigou	10	10 / 182	5.5
>4000BP	4500–4000	Kangjia	16	42 / 146	28.8
>4000BP	4500–3900	Taosi	180	28 / 2895	1.0
<4000BP	4000–3600	Sanguan	7	8 / 181	4.4
<4000BP	3900–3500	Donglongshan	24	35 / 244	14.3
<4000BP	3100–2800	Nianzipo	111	199 / 1739	11.4
<4000BP	3100–2800	Shaolingyuan	147	220 / 1760	12.5
<4000BP	3100–2800	Zhouyuan	25	63 / 677	9.3
<4000BP	3100–2800	Qucun	25	33 / 483	6.8
<4000BP	3100–2000	Dongyang	47	55 / 726	7.6
<4000BP	2800–2500	Yanqing	171	341 / 3767	6.5
<4000BP	2800–2500	Shangma	130	166 / 2548	9.1
<4000BP	2500–2200	Zhaitouhe	23	38 / 502	7.6
<4000BP	2500–2200	Qujia Wanlijia	83	241 / 1199	20.1
<4000BP	2500–2000	Qiaocun	514	793 / 6954	11.4
<4000BP	2200–2000	Podi	111	51 / 1633	3.1
<4000BP	2000–1750	Xitun	201	256 / 3784	6.8
** **	** **	**GQ Sites**	** **	** **	
>4000BP	4400–4000	Liuwan	53	22 / 793	2.8
<4000BP	4000	Mogou	223	298 / 4893	6.1
<4000BP	3000–2200	Xishan	22	39 / 364	10.7

Prevalence of dental caries data in a population is usually studied with reference to a host of other information, most importantly demographic compositions, as the occurrence of dental caries varies in individuals of different age and sex. Unfortunately, due to a lack of standardization in data reporting, an in-depth analysis is not possible in this study. While we recognize the lack of demographic information has posed severe limitations to the power of our analysis, an assessment of dental caries data is still included to provide additional measures to underscore changes in ancient Chinese subsistence strategies. As such, we are not trying to make detailed inferences about the pattern of dental caries rate among these populations. Instead, the result of dental caries analysis should be treated as a broad-stroke trend analysis to complement our stable isotopic data. Furthermore, for sites that have accompanying demographic information, a vast majority of them have very similar demographic compositions (for details please see S2 File- Supporting Information). Therefore, a general average from each site should be sufficiently representative for the estimation of a general tendency of dental caries prevalence at each site.

## 4. Results

### 4.1 Stable isotope analysis

**Fig 2:**
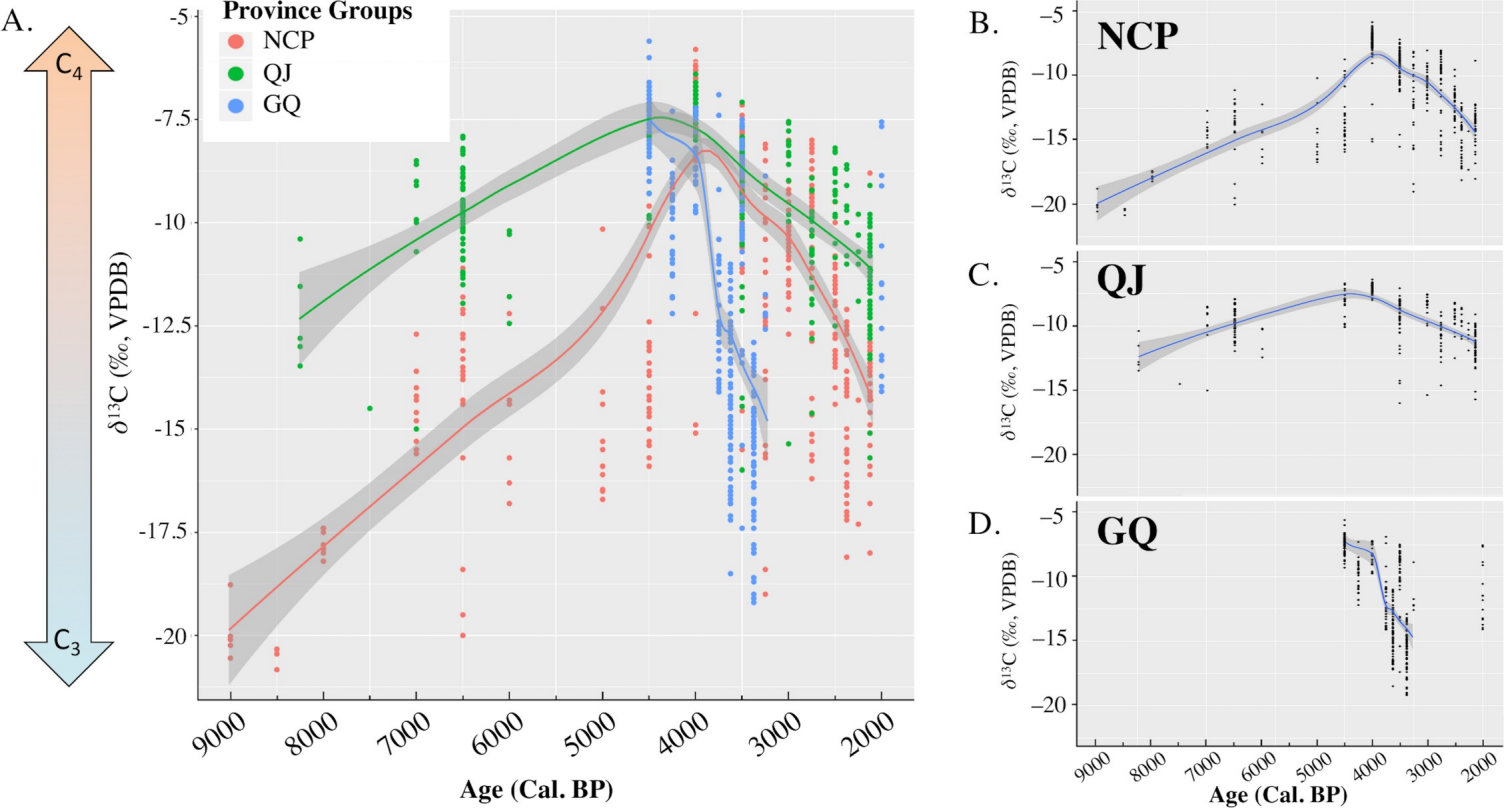
Bone collagen *δ*^13^C values of humans from all sites discussed in this study, organized by province groups. Note that due to the 1000-year gap between the last two groups of data from the GQ region, the smooth line was not extended to the last group.

The *δ*^13^C values of all individuals analysed are plotted in Fig 2 and summarized in Table 3. Considering exclusive C_4_ consumers can have *δ*^13^C values between –13.1‰ and –3.7 ‰, and exclusive C_3_ feeders between –28.1‰ and –14.7 ‰ [131–133], our data demonstrate a marked increase in the proportion of C_4_ foodstuffs consumed across northern China (both NCP and QJ regions) through 9000 to 4000 BP. This dependency on C_4_ foodstuffs peaked at around 4000–3500 BP in both regions in northern China (NCP: –7.3±1.4‰, n = 96; QJ: –7.1±0.3‰, n = 62). Subsequently, the importance of C_3_ crops began to grow, signified by the increasingly depleted *δ*^13^C values of residents across all regions. By the end of the study period, groups in NCP and QJ have mean *δ*^13^C values of –13.9±1.5‰ (n = 42) and –11.5±1.3‰ (n = 48), respectively. In GQ, despite a lack of isotopic data prior to 4500 BP, our data from 7 sites (–8.0 ± 1.4‰, n = 99) heavily imply that during the 4500–4000 BP period, groups in this region still subsisted largely on C_4_ crops, but the importance of C_4_ foodstuffs began to wane after this period. By 3000 BP, groups in GQ have a mean *δ*^13^C value of (–13.0±3.5‰, n = 131).

### 4.2 Dental caries analysis

**Fig 3:**
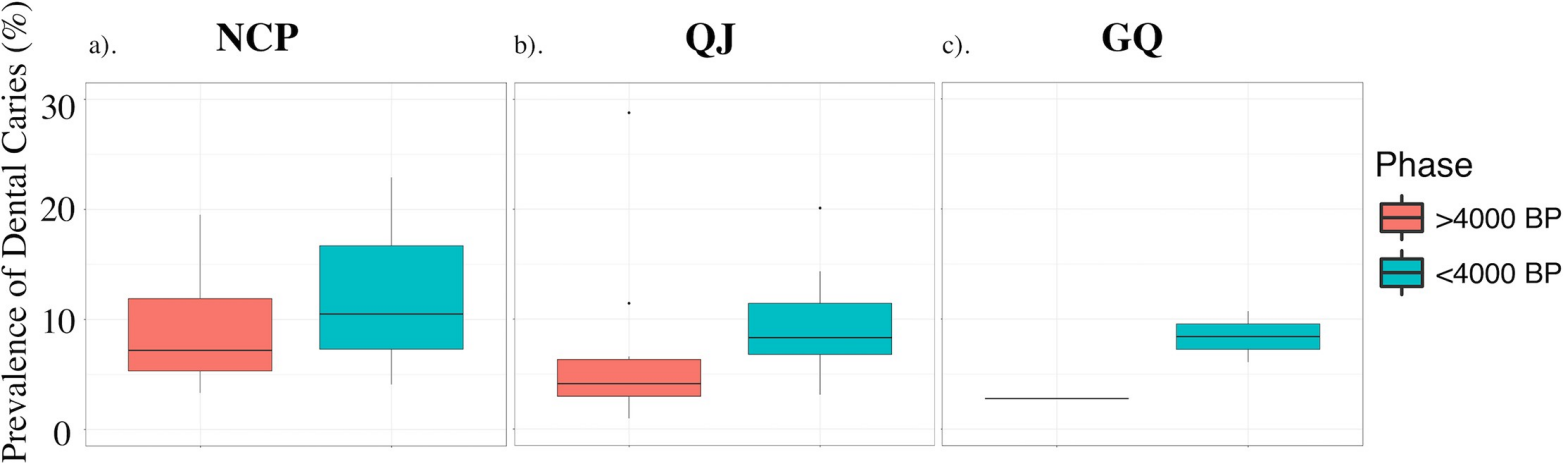
Boxplots showing changes in the prevalence of dental caries before and after 4000 BP in the three regions: a) changes in prevalence of dental caries in NCP; b) changes in prevalence of dental caries in QJ; c) changes in prevalence of dental caries in GQ.

Data for the rates of dental caries in the three regions are plotted in Fig 3 and summarized in Table 4. Summary statistics are shown in Table 5. Among all regions, groups from NCP reported the largest variability in both the pre- and post- 4000BP phases, with IQR ranges of 5.3–11.9% and 7.3–16.7%, respectively. Overall, the prevalence of dental caries has increased substantially over time across all three regions.

**Table 5:** Summary statistics of the dental caries data (%).

Phase	Region	Minimum value	1^st^ quartile	Median	3^rd^ quartile	Maximum value
**Pre-4000 BP**	NCP	3.3	5.3	7.2	11.9	19.5
**Post-4000 BP**		4.1	7.3	10.5	16.7	22.9
**Pre-4000 BP**	QJ	2.6	3.4	4.3	6.6	28.8
**Post-4000 BP**		1.0	6.6	7.6	11.4	20.1
**Pre-4000 BP**	GQ	2.8	2.8	2.8	2.8	2.8
**Post-4000 BP**		6.1	6.1	8.4	10.7	10.7

## 5. Discussion

### 5.1 Stable isotope analysis

Between 9000–4500 BP, the enrichment in mean *δ*^13^C values across northern China (NCP and QJ) likely reflects the last stages of “Neolithic revolution” in China, where full-fledged agriculture was becoming the primary subsistence economy of groups in these regions [126, 134]. During this period, the mean *δ*^13^C values of the NCP and QJ rose from –19.9±0.7 ‰ to –12.9±2.2 ‰, and –12.2±1.3 ‰ to –8.3±1.1 ‰, respectively. As discussed earlier, millet, a C_4_ crop, was the first, as well as the most significant domesticated crop in Neolithic China [23, 27]. Thus, the changes in *δ*^13^C values of these northern Chinese communities fit nicely with this narrative, confirming the increasing reliance on millet agriculture among these communities during this period. Interestingly, both the Yiluo River Valley 伊洛河流域 (NCP) and Wei River Basin 渭河流域 (QJ) are long regarded as the birthplace of millet agriculture in northern China [23, 28, 35, 83, 135]. However, as demonstrated by Fig 2, sites in QJ relied much more heavily on millet than sites in NCP through 8000–4000 BP. The earliest evidence of domesticated millet in China so far is from Nanzhuangtou 南莊頭, a Neolithic site in NCP dated to 11,500–11,000 cal. BP [28]. Our results may suggest that despite being domesticated in NCP first, millet was consumed in considerably higher proportions in QJ throughout the late Neolithic to early Bronze Age periods. Furthermore, evidence of mixed farming of millet and rice (a C_3_ crop) was found in many NCP sites, mostly in Shandong, such as Xihe 西河 (ca. 8000 cal. BP), Yuezhuang 月莊 (8019–7700 cal. BP), Dongpan 東盤 (5980–5870 cal. BP), and also Tanghu 唐戶 in Henan (7800–4500 cal. BP) [32, 35, 136, 137]. While there has not been any isotopic work published on these particular sites, the mean *δ*^13^C values of humans from Xiaojingshan, a site contemporaneous with, and culturally related to Yuezhuang and Xihe, is –17.8.3 ± 0.3‰ (n = 10)[116]. This is consistent with values reported from groups subsisted on a mixed C_3_/C_4_ diet. Thus, our data suggested that during the 9000–4500 BP period, groups in QJ generally had a more focused agricultural strategy compared to their contemporaries in NCP. In NCP, the overall agricultural strategy also became increasingly focused over time, but never to the extent displayed in QJ.

The highest mean *δ*^13^C values in each respective region are –7.3±1.4 ‰ in NCP (4000–3500 BP), –7.1±0.3 ‰ in QJ (4000–3500 BP), and –8.0±1.4 ‰ in GQ (4500–4000 BP) (see Table 3). During these periods, groups in these regions likely subsisted primarily on C_4_ crops (e.g. millet) and their subsidiary products, and thus possibly reflected the height of a millet-dependent economy during this period in these regions. However, this changed very quickly as shown by the isotopic “turning points” in Fig 2. First in GQ and QJ, then in NCP, C3 –based foods were becoming increasingly important in these regions. While archaeological evidence suggests that some communities, such as the Siwa Culture (GQ; ca. 3300–2500 BP), switched to a mixed pastoral-agricultural economy [5, 138, 139], it is more likely that C_3_ crops such as wheat, barley, and rice were gaining in popularity among most other communities. This is supported by palaeobotanical evidence from many different sites. A recent study reviewing and analysing palaeobotanical data from 45 sites in the Haidai region (NCP–Shandong province) observed the following trend among carbonized cereal grains: around 8% of the grains from Longshan period sites (5000–4000 BP) were wheat; around 12% of the grain from Shang period sites (4000–3000 BP) were wheat; and over 58% of grains from East and West Zhou dynasties sites (3000–2000BP) were wheat [41]. Similar trends were also observed in sites in the Luoyang Basin (NCP–Henan province) [140].

Nevertheless, by the end of this study period (1750 BP), the mean *δ*^13^C values of the three regions during these phases are –13.9±1.5 ‰, –11.5±1.3 ‰, and –11.4±2.3 ‰, respectively (see Table 3). The net shift in *δ*^13^C values (NCP: +6.6‰, QJ: +4.4‰, GQ:+3.4‰) suggests that by 1750 BP, a greater proportion of C_3_ foodstuffs was being consumed. This indicates that there were considerable changes in subsistence economies across all regions between the period of 4000–1750 BP. Unfortunately, without archaeological faunal and botanical isotopic baselines from each site, it is not possible to quantify the changes in the proportion of C_3_– vs. C_4_– based food among the groups analysed in this study.

### 5.2 Dental caries analysis

#### 5.2.1 Pre-4000 BP phase

During the pre-4000 BP phase, the prevalence of dental caries was most variable in NCP. This is probably due to the more favourable climatic conditions in this region, where a wider range of resources were available to the inhabitants, including starchy, and therefore highly cariogenic plants such as various roots and tubers, acorn, and Job’s tears [141–143]. A recent study evaluated the temporal changes in tool assemblage from the site Jiahu (one of the NCP sites examined in this paper), concluding that while tools related to farming did increase over time, the assemblage was dominated (~75%) by tools related to hunting and gathering [144]. Note that native plants in northern China are overwhelmingly C_3_ plants [145]. As the two phases of Jiahu communities have a mean *δ*^13^C value of –20.2 ± 0.6‰ (n = 9) [109], the stable isotopic and dental caries data both concur that during the beginning of our study period, most communities in NCP likely still relied heavily on foraged food for subsistence, despite having an earlier start in the domestication of millet.

The frequencies of dental caries were markedly lower in QJ and GQ during this period. A recent palaeobotanical study on an early Yangshao Culture site (c.7000–6000 BP), Yuhuazhai (QJ), revealed that other than millet, the most abundant plant taxa was *Chenopodium*, a genus that includes several edible leafy species such as goosefoot (*C*. *album*) and tree spinach (*C*. *giganteum*) [146, 147]. Similar findings from another ethnobotanical study cataloguing edible wild plant species available in the two valleys within the Qinling Mountains (within QJ) also reported a high number of leafy vegetable species, including (but not limited to) the *Chenopodium* varieties [148]. Thus, we propose that the lower prevalence of dental caries in this region possibly reflected differences in QJ’ and NCP’s plant communities, where the former is characterised by a higher number of edible leafy vegetables, and the latter by more starchy plant foods. Nonetheless, most of the wild vegetables found in QJ, including many species from the genus *Chenopodium*, are C_3_ plants. Thus, the enriched *δ*^13^C values (Fig 2) of the inhabitants in QJ indicate that C_4_ crops, such as millet, were still a major contributor to the dietary intake of the QJ inhabitants, and thus millet agriculture must be already well-established in this region as early as 8000 BP.

The situation in GQ during the pre-4000 BP phase is harder to tease out, as we only have data from one site, Liuwan. Liuwan belonged to the Majiayao Culture, which consisted of groups subsisting on a mixed pastoral-agricultural economy living across part of the modern provinces of Gansu, Qinghai, and northern Sichuan [149]. The low occurrence of dental caries in this site (2.8%) is in line with the lifeways of other archaeological agro-pastoral populations [105, 150–153].

#### 5.2.2 Post-4000 BP phase

In the post-4000 BP phase, just as the trend in *δ*^13^C values revealed a sudden change in subsistence economy across the landscape, the prevalence of dental caries across all three regions also increased substantially (Fig 3). Since the middle Neolithic period, agriculture spread rapidly across northern and northwestern China rapidly, as shown by the increasingly elevated *δ*^13^C values of groups in both NCP and QJ throughout 9000–4500 BP (Fig 2). While earlier sweeping changes in *δ*^13^C values were likely brought about by the spread of (millet) agriculture, the increase in prevalence of dental caries in the post-4000 BP period may signify an increasing reliance on agricultural products. As the IQR ranges and medians of the prevalence of dental caries in these three regions all fall within the range presented by other studies for agricultural societies (2.1% - 26.9%) [11, 154], we propose that this substantial increase in the occurrence of dental caries was a result of an increasing reliance on carbohydrates as a food source. This trend is particularly strong in GQ, where the sharp increase in the occurrence of caries (Fig 3) indicates substantial changes in dietary composition. During the late Neolithic period, intensive rain-fed agriculture was introduced into northwestern China from the east [27, 155]; around the same time, energy-rich carbohydrates such as wheat and barley were brought in from Central Asia [18, 31]. The growing availability and consumption of these starchy cereals could therefore contribute to a higher level of dental caries at the population level. This is corroborated by numerous studies in other parts of the world, reporting a similarly marked increase in the prevalence of dental caries among populations that has switched subsistence economy from mixed to fully agricultural [154, 156]. Hence, the magnitude of changes in the prevalence of dental caries in GQ (Fig 2) likely represents the progressively more important role of agriculture among many of these communities.

Meanwhile in NCP and QJ, the accompanying drop in *δ*^13^C values and increase in the prevalence of dental caries likely suggests that in these regions, agricultural practices both intensified and diversified. The intensification of agricultural practices can be attested by the increase in prevalence of dental caries, likely caused by a higher intake of carbohydrates among these groups (Fig 3). While the diversification of subsistence economy is reflected in the dropping of mean *δ*^13^C values, which shows that a wider variety of C_3_ crops, such as wheat and barley, were grown to supplement millet production. Unfortunately, out of the 77 sites discussed, we only have 11 sites with overlapping stable isotopic and dental caries data. The paucity of sites with overlapping data types has prevented more thorough investigation of this phenomenon. While we elect to not make any further analytical conclusions from these 11 sites, the list of these sites and the summary statistics of both stable isotopic and dental caries data are presented in S2 File–Supporting Information for interested readers.

Despite the fact that sites in all three regions exhibit higher prevalence of dental caries in the post-4000 BP phase, the medians of prevalence rate (2.8%, 7.6%, and 8.4%, respectively) are still much lower than populations that subsisted primarily on refined carbohydrates (>30%) [157]. Furthermore, there are large variations in the prevalence of dental cares among some fully agricultural groups in the same region. For example, people from Yinxu [111, 112], and Taosi [76] possess very low rates of dental caries (4.1% and 1.0%, respectively), while those from Chenjiagou [105] demonstrate a much higher prevalence of dental caries (22.9%). While the overrepresentation of elderly individuals in Chenjiagou may explain this abundance of caries [105], there are a number of other possible explanations for this variability. For example, variations in enamel thickness, presence or absence of certain enamel formation genes, saliva type and quantity, natural levels of fluorine or other minerals in water, and overall health status during the growth and development of teeth, can influence caries susceptibility in different populations [158–162]. While it is not possible to practically to account for all these factors in a meta-data analysis, we encourage readers interested in understanding the occurrence of dental caries in any particular sites to consider examining those variants. One other possible explanation for the markedly varied prevalence of dental caries among fully agricultural groups could be due to differing food preparation techniques [13]. As mentioned earlier, during this time, most cereals, including wheat, were likely consumed as gruel. Unrefined cereals are very gritty, and therefore would be less cariogenic than refined cereal products [163, 164]. Therefore, it is possible that the large discrepancies in the occurrence of dental caries reflect different cereal processing methods among these different communities.

Furthermore, palaeobotanical evidence indicates that wheat has arrived in northern China as early as the late Neolithic period. However, according to our isotopic data, by the end of our study period (c. 1750 BP), wheat and other C_3_ crops were still a relatively minor component in most groups’ diets. Historical sources suggested that more advanced wheat processing techniques likely only become prevalent during the Tang Dynasty. The unpalatable nature of wheat gruel meant that early in its adoption, wheat was likely taken as a substitute for millet during difficult times. Thus, this suggested that the major impediment to the popularization of wheat in northern China could be the lack of processing techniques to improve palatability and digestibility, a development that lagged behind the initial availability of the grain.

Unfortunately, we do not have corresponding archaeological evidence from each site to corroborate our argument yet. To further test this hypothesis, additional, more granular data about subsistence patterns is required.

### 5.3 The catalyst

An interesting feature present in our data are the isotopic “turning points” occurring at around 4500–4000 BP in QJ and 4000–3500 BP in NCP Fig 2). These almost simultaneous turning points indicate a marked shift in the subsistence economies in groups across all three regions during this period, where groups have changed from an almost exclusively C_4_– based diet, to a diet also encompassing variable quantities of C_3_ foods. While it is quite possible that the “turning point” is an artifact of the patchiness of the isotopic records in some phases, there are several alternative explanations. Sudden changes in subsistence strategies often result from a myriad of cascading factors, such as climatic or ecological change, population growth, and socio-political upheaval [165–169]. The development of agriculture allowed exponential population growth in northern and northwestern China, especially towards the end of the Neolithic period [170]. However, rapid population growth alone cannot explain the almost simultaneous, cross-cultural, cross-regional change in subsistence economies as shown in Fig 2. A larger, and more urgent catalyst is needed to drive this shift. One possibility is climate change. In fact, our isotopic turning points coincide with the “Holocene Event 3”–a global aridification event that occurred at around 4200 BP [138, 171–173]. Archaeologists have long observed intense social upheavals in response to this event across all continents, including the collapse of the Akkadian empire in Mesopotamia [174], the collapse of Liangzhu Culture in the lower Yantze River valley in China [175], and many more [176]. Thus, it is possible that the trend moving from a narrowly focused, mainly millet-based diet, to a more diverse agricultural system was instigated by this climatic event.

A crucial question remains, however, that in the face of increased aridity, how, and why did the ancient Chinese farmers turn to wheat? Wheat has a higher water requirement than millet, which makes it a poorer contestant than drought-resistant millet. Thus, we propose that climate change did not prompt groups in northern China to grow wheat in order to simply replace millet. Instead, among all other incoming crops, wheat was selected specifically to complement millet agriculture in response to resources shortages, brought on by a number of factors, including abrupt climate change. As mentioned earlier, wheat, or winter wheat, can be sowed during winter after millet has been harvested. During periods of fluctuating climate (hence attendant fluctuation in crop yield) and rapid population growth, alternating sowing and harvesting seasons of millet and wheat can ensure consistent year-round crop production, and thus provide an invaluable additional food source. The utilization of the difference in winter wheat sowing time is in fact well documented in historical sources. According to inscriptions on oracle bones found in the late Shang site Yinxu, “Zheng” month (正月), which is roughly April or May in the Gregorian calendar, was the time for wheat harvesting [177]. This suggests that seeds were sown during winter. Other sources also reported sowing wheat in hard winters during the Han Dynasties [178]. Moreover, the Erlitou culture/ Xia Dynasty—long considered to be China’s first state-level society—was founded at around 3900 BP. Since then, many other complex state-societies have emerged in northern and northwestern China [179]. Thus, the subpar growing conditions for wheat during this period could be mitigated by the increasingly centralized government control of large-scale infrastructure (i.e. irrigation systems). For example, some of the world’s earliest hydraulic engineering works in water management are seen in Han Dynasty China [180, 181]. Thus, despite the less than ideal climatic conditions, any extra yield obtained through the incorporation of winter wheat into a crop rotation system would be a bonus to the people who were initially relying on only millet. As a result, wheat was likely used to supplement millet production. Other crops, such as barley, soybean, and depending on region, rice, were also grown in varying extent to alleviate pressure on demand for food resources, as demonstrated in the increasingly diversified palaeobotanical remains in later sites [31, 39]. Hence, the increasing consumption of C_3_ food and the elevated prevalence of dental caries that characterizes 4000 BP through to 1750 BP across all regions may potentially reflect a diversification in ancient Chinese subsistence economy as a response to an abrupt climatic event.

## 6. Conclusion

Bringing together existing isotopic and dental pathology data, our study has shed light onto the process of how subsistence economies diversified in groups across northern and northwestern China over a period of 7250 years. Our results demonstrate that a diachronic meta-data analysis can reveal immensely valuable clues about past societies. Firstly, the closely timed isotopic turning points across the three geographic regions provide new insight into the manner in which introduced crops were adopted across the landscape. Even though we could not quantify the absolute percentage of C_3_– vs C_4_– based food consumed in each region due to the lack of local faunal and floral baseline data, the numbers nonetheless provided a proxy for us to understand temporal changes in subsistence economies. Secondly, our data also suggests that the increasingly intense population pressure as well as fluctuating climatic conditions across China during the late Neolithic period drove groups towards more a more effective and diversified subsistence economy.

However, despite being a very powerful analytical tool, it is also important to be aware of some of the pitfalls associated with meta-analyses. One of the most significant pitfalls is that meta-analysis does not permit a fine-tuned analysis of the data. In this particular study, the ultimate replacement of millet by wheat as the staple crop of northern China should not be seen as a simple linear process–the advantage of wheat over millet was probably not apparent to the ancient people initially. This is evident in the large intra-site variations in *δ*^13^C values as well as prevalence of dental caries across all regions. Thus, as suggested by several other studies, the manner in which a new crop is accepted by a population needs to be understood within a complex matrix of ecological, socio-political, and cultural factors [5, 65]. While it is beyond the scope of this study to discuss the particularities of each individual site, we urge future studies to partake in deeper investigations into these sites, to better understand the impacts of social institutions on subsistence strategies. Finally, we hope more isotope and dental caries data from the post-Han periods will become available in the near future. Currently, most isotopic and population health research in China tends to focus on early prehistoric periods, and pays very little attention to sites from the historical periods. The incorporation of additional data from these later periods will allow us to cross check our findings with historical texts, and ultimately allow us to better evaluate the persistence of wheat consumption in China during the historical periods.

## Supporting information

S1 File R scripts. (DOCX)

S2 File Supplementary information. Principles and caveats of analytical techniques.
(DOCX)

S3 File Isotope data. Detailed sample information, and the bone collagen *δ*^13^C and *δ*^15^N values of all samples analyzed in this study. The column “Radiocarbon date” refers to whether the site has been radiocarbon dated: Y indicates that radiocarbon dates are provided in the cited studies/study; N indicates that no radiocarbon date is provided in the cited studies/study.
(XLSX)

S4 File Dental caries data. Detailed site information and the prevalence of dental caries (%) of all sites analyzed in this study.
(XLSX)
