# Supplementary material for: Stable isotope and dental caries data reveal abrupt changes in subsistence economy in ancient China in response to global climate change
Source: PLoS One. 2019 Jul 22;14(7):e0218943. doi: 10.1371/journal.pone.0218943 (PMC6645670; doi:10.1371/journal.pone.0218943)
Supplement: S1 File — (DOCX) [file pone.0218943.s001.docx]

R scripts used in this study

### for isotopic analysis ###

attach(Isotope_R)

library(ggplot2)

NCP <- subset(Isotope_R, Province_group=="1")

QJ <- subset(Isotope_R, Province_group=="2")

GQ <- subset(Isotope_R, Province_group=="3")

# this is to exclude the last site with the 1000-year-gap

GQ2 <- subset(GQ, Phase!="15")

# scatterplot of regional data with local regression line

#for carbon in NCP

ggplot(NCP, aes(x=Phase, y=d13C))+geom_point()+geom_smooth()+

labs(x="Age (Cal.BP)", y=expression(paste(delta^13,"C (\u2030, VPDB)")))+

scale_y_continuous(limits=c(-22.5,-5))+scale_x_continuous(limits=c(0,15))

#for carbon in QJ

ggplot(QJ, aes(x=Phase, y=d13C))+geom_point()+geom_smooth()+

labs(x="Phase", y=expression(paste(delta^13,"C (\u2030, VPDB)")))+

scale_y_continuous(limits=c(-22.5,-5))+scale_x_continuous(limits=c(0,15))

#for nitrogen in NCP

ggplot(NCP, aes(x=Phase, y=d15N))+geom_point()+geom_smooth()+

labs(x="Phase", y=expression(paste(delta^15,"N (\u2030, AIR)"))) +

scale_y_continuous(limits=c(4,16))+scale_x_continuous(limits=c(0,15))

#for nitrogen in QJ

ggplot(QJ, aes(x=Phase, y=d15N))+geom_point()+geom_smooth()+

labs(x="Phase", y=expression(paste(delta^15,"N (\u2030, AIR)"))) +

scale_y_continuous(limits=c(4,16))+scale_x_continuous(limits=c(0,15))

#for GQ, due to the gap in the data, the regression line is produced separately

#for carbon in GJ

ggplot(GQ, aes(x=Phase, y=d13C))+geom_point()+geom_smooth()+

labs(x="Phase", y=expression(paste(delta^13,"C (\u2030, VPDB)")))+

scale_y_continuous(limits=c(-22.5,-5))+scale_x_continuous(limits=c(0,15))

p1<-ggplot(GQ2, aes(x=Phase, y=d13C))+geom_smooth()+

labs(x="Phase", y=expression(paste(delta^13,"C (\u2030, AIR)")))+

theme(

panel.background = element_rect(fill = "transparent")

, plot.background = element_rect(fill = "transparent")

, panel.grid.major = element_blank()

, panel.grid.minor = element_blank()

, legend.background = element_rect(fill = "transparent")

, legend.box.background = element_rect(fill = "transparent"))

ggsave(p1, filename = "Rplot_smoothline_GQ_d13C.png", bg = "transparent")

#for nitrogen in GQ

ggplot(GQ, aes(x=Phase, y=d15N))+geom_point()+geom_smooth()+

labs(x="Phase", y=expression(paste(delta^15,"N (\u2030, AIR)"))) +

scale_y_continuous(limits=c(4,16))+scale_x_continuous(limits=c(0,15))

p2<-ggplot(GQ2, aes(x=Phase, y=d15N))+geom_smooth()+

labs(x="Phase", y=expression(paste(delta^15,"N (\u2030, AIR)")))+

theme(

panel.background = element_rect(fill = "transparent")

, plot.background = element_rect(fill = "transparent")

, panel.grid.major = element_blank()

, panel.grid.minor = element_blank() =

, legend.background = element_rect(fill = "transparent")

, legend.box.background = element_rect(fill = "transparent"))

ggsave(p2, filename = "Rplot_smoothline_GQ_d15N.png", bg = "transparent")

# for all three regions

ggplot(Isotope_R, aes(x=Phase, y=d13C, color=factor(Province_group)))+geom_point()+

labs(x="Phase", y=expression(paste(delta^13,"C (\u2030, VPDB)"))) + scale_y_continuous(breaks=seq(-22.5,-5))

p3<-ggplot(NCP, aes(x=Phase, y=d13C))+geom_smooth()+

labs(x="Phase", y=expression(paste(delta^13,"C (\u2030, AIR)")))+

theme(

panel.background = element_rect(fill = "transparent")

, plot.background = element_rect(fill = "transparent")

, panel.grid.major = element_blank()

, panel.grid.minor = element_blank()

, legend.background = element_rect(fill = "transparent")

, legend.box.background = element_rect(fill = "transparent"))

ggsave(p3, filename = "Rplot_smoothline_NCP_d13C.png", bg = "transparent")

p4<-ggplot(QJ, aes(x=Phase, y=d13C))+geom_smooth()+

labs(x="Phase", y=expression(paste(delta^13,"C (\u2030, AIR)")))+

theme(

panel.background = element_rect(fill = "transparent")

, plot.background = element_rect(fill = "transparent")

, panel.grid.major = element_blank()

, panel.grid.minor = element_blank()

, legend.background = element_rect(fill = "transparent")

, legend.box.background = element_rect(fill = "transparent"))

ggsave(p4 filename = "Rplot_smoothline_QJ_d13C.png", bg = "transparent")

p5<-ggplot(GQ2, aes(x=Phase, y=d13C))+geom_smooth()+

labs(x="Phase", y=expression(paste(delta^13,"C (\u2030, AIR)")))+

theme(

panel.background = element_rect(fill = "transparent")

, plot.background = element_rect(fill = "transparent")

, panel.grid.major = element_blank()

, panel.grid.minor = element_blank()

, legend.background = element_rect(fill = "transparent")

, legend.box.background = element_rect(fill = "transparent"))

ggsave(p5, filename = "Rplot_smoothline_GQ_d13C.png", bg = "transparent")

# scatterplot of data by sites

##carbon

ggplot(NCP, aes(x=Phase, y=d13C, color=Site))+geom_point()+

labs(x="Phase", y=expression(paste(delta^13,"C (\u2030, VPDB)"))) +

scale_y_continuous(limits=c(-22.5,-5))+scale_x_continuous(limits=c(0,15))

ggplot(QJ, aes(x=Phase, y=d13C, color=Site))+geom_point()+

labs(x="Phase", y=expression(paste(delta^13,"C (\u2030, VPDB)"))) +

scale_y_continuous(limits=c(-22.5,-5))+scale_x_continuous(limits=c(0,15))

ggplot(GQ, aes(x=Phase, y=d13C, color=Site))+geom_point()+

labs(x="Phase", y=expression(paste(delta^13,"C (\u2030, VPDB)"))) +

scale_y_continuous(limits=c(-22.5,-5))+scale_x_continuous(limits=c(0,15))

##nitrogen

ggplot(NCP, aes(x=Phase, y=d15N, color=Site))+geom_point()+

labs(x="Phase", y=expression(paste(delta^15,"N (\u2030, AIR)")))+

scale_y_continuous(limits=c(4,16)) +scale_x_continuous(limits=c(0,15))

ggplot(QJ, aes(x=Phase, y=d15N, color=Site))+geom_point()+

labs(x="Phase", y=expression(paste(delta^15,"N (\u2030, AIR)")))+

scale_y_continuous(limits=c(4,16)) +scale_x_continuous(limits=c(0,15))

ggplot(GQ, aes(x=Phase, y=d15N, color=Site))+geom_point()+

labs(x="Phase", y=expression(paste(delta^15,"N (\u2030, AIR)")))+

scale_y_continuous(limits=c(4,16)) +scale_x_continuous(limits=c(0,15))

### for dental caries ###

attach(Caries_R)

library(ggplot2)

NCP <- subset(Caries_R, Province=="1")

QJ <- subset(Caries_R, Province=="2")

GQ <- subset(Caries_R, Province=="3")

# for the box plots #

bp1 <- ggplot(NCP, aes(x=as.factor(Phase), y=percentage, group=Phase, fill=as.factor(Phase)))+geom_boxplot() +

theme_bw() + labs(x="Phase", y="Prevalence of Dental Caries (%)") +

scale_y_continuous(limits=c(0,30))+

scale_x_discrete(labels=c("1"=">4000 BP", "2"="<4000 BP")) +

scale_fill_discrete(name="Phase", labels=c(">4000 BP", "<4000 BP"))

bp2<- ggplot(QJ, aes(x=as.factor(Phase), y=percentage, group=Phase, fill=as.factor(Phase)))+geom_boxplot()+

theme_bw() + labs(x="Phase", y="Prevalence of Dental Caries (%)") +

scale_y_continuous(limits=c(0,30))+

scale_x_discrete(labels=c("1"=">4000 BP", "2"="<4000 BP")) +

scale_fill_discrete(name="Phase", labels=c(">4000 BP", "<4000 BP"))

bp3 <-ggplot(GQ, aes(x=as.factor(Phase), y=percentage, group=Phase, fill=as.factor(Phase)))+geom_boxplot()+

theme_bw() + labs(x="Phase", y="Prevalence of Dental Caries (%)") +

scale_y_continuous(limits=c(0,30))+

scale_x_discrete(labels=c("1"=">4000 BP", "2"="<4000 BP")) +

scale_fill_discrete(name="Phase", labels=c(">4000 BP", "<4000 BP"))

##Chi-square tests

#NCP

Affected.NCP = c(292, 332)

Absent.NCP = c(3228, 2885)

NCP.chisq= as.data.frame(rbind(Affected.NCP,Absent.NCP))

names(NCP.chisq) = c(">4000BP","4000BP")

chisq.test(NCP.chisq, correct=FALSE)

#QJ

Affected.QJ = c(231, 2499)

Absent.QJ = c(6342, 23698)

QJ.chisq= as.data.frame(rbind(Affected.QJ,Absent.QJ))

names(QJ.chisq) = c(">4000BP","4000BP")

chisq.test(QJ.chisq, correct=FALSE)

#GQ

Affected.GQ = c(320, 39)

Absent.GQ = c(5366, 325)

GQ.chisq= as.data.frame(rbind(Affected.GQ,Absent.GQ))

names(GQ.chisq) = c(">4000BP","4000BP")

chisq.test(GQ.chisq, correct=FALSE)

#Total

Affected.total = c(843, 2870)

Absent.total = c(14936, 26908)

total.chisq= as.data.frame(rbind(Affected.total,Absent.total))

names(total.chisq) = c(">4000BP","4000BP")

chisq.test(total.chisq, correct=FALSE)

#Tukey 5 number summary

#x.1 = min value, x.2= 1st quartile, x.3 = median, x.4=3rd quartile, x.5=max value

#group 1= NCP phase 1, group 2 = NCP phase 2, group 3 = QJ phase 1, group 4 = QJ phase 2, group 5 = GQ phase 1, group 6 = GQ phase 2

summary <- aggregate(percentage, by=list(Group), fivenum)

# by sex

NCP_S <- subset(NCP, Sex=="1"|Sex=="2")

QJ_S <- subset(QJ, Sex=="1"|Sex=="2")

GQ_S <- subset(GQ, Sex=="1"|Sex=="2")

#by age

NCP_A <- subset(NCP, Age=="1"|Age=="2"|Age=="3")

QJ_A <- subset(QJ, Age=="1"|Age=="2"|Age=="3")

GQ_A <- subset(GQ, Age=="1"|Age=="2"|Age=="3")

#for the histograms#

#by sex

hist1 <- ggplot(NCP_S, aes(Site, sex_percentage, fill=as.factor(Sex))) +geom_bar(stat="identity", position="dodge")+

scale_fill_discrete(name="Sex", labels=c("Male", "Female"))+

labs(x="Site", y="Prevalence of Dental Caries (%)")+

scale_y_continuous(limits=c(0,35))

hist2 <- ggplot(QJ_S, aes(Site, sex_percentage, fill=as.factor(Sex))) +geom_bar(stat="identity", position="dodge")+

scale_fill_discrete(name="Sex", labels=c("Male", "Female"))+

labs(x="Site", y="Prevalence of Dental Caries (%)")+

scale_y_continuous(limits=c(0,35))

hist3 <- ggplot(GQ, aes(Site, sex_percentage, fill=as.factor(Sex)))

+geom_bar(stat="identity", position="dodge")+

scale_fill_discrete(name="Sex", labels=c("Male", "Female"))+

labs(x="Site", y="Prevalence of Dental Caries (%)")+

scale_y_continuous(limits=c(0,35))

#by age

hist4 <- ggplot(NCP_A, aes(Site, age_percentage, fill=as.factor(Age))) +geom_bar(stat="identity", position="dodge")+

scale_fill_discrete(name="Age Group", labels=c("Subadult", "Young Adult", "Old Adult"))+

labs(x="Site", y="Prevalence of Dental Caries (%)")+

scale_y_continuous(limits=c(0,35))

hist5 <- ggplot(QJ_A, aes(Site, age_percentage, fill=as.factor(Age))) +geom_bar(stat="identity", position="dodge")+

scale_fill_discrete(name="Age Group", labels=c("Subadult", "Young Adult", "Old Adult"))+

labs(x="Site", y="Prevalence of Dental Caries (%)")+

scale_y_continuous(limits=c(0,35))

hist6 <- ggplot(GQ, aes(Site, age_percentage, fill=as.factor(Age)))

+geom_bar(stat="identity", position="dodge")+

scale_fill_discrete(name="Age Group", labels=c("Subadult", "Young Adult", "Old Adult"))+

labs(x="Site", y="Prevalence of Dental Caries (%)")+

scale_y_continuous(limits=c(0,35))

#by period and site

#first further subset data by phase

NCP.1 <-subset(NCP, Phase=="1")

NCP.2 <-subset(NCP, Phase=="2")

QJ.1 <-subset(QJ, Phase=="1")

QJ.2 <-subset(QJ, Phase=="2")

GQ.1 <-subset(GQ, Phase=="1")

GQ.2 <-subset(GQ, Phase=="2")

# manually enter the order of entries by frequency #

#reorder NCP Phase 1

NCP.1$Site <-factor(NCP.1$Site, levels =c("Dawenkou", "Xiawanggang", "Miaodigou", "Gouwan", "Duzhong", "Qinglongquan", "Guangwu"))

#then plot

ggplot(NCP.1, aes(Site, percentage))+ geom_bar(stat="identity", fill="#F8766D")+

labs(x="Site", y="Prevalence of Dental Caries (%)") +

scale_y_continuous(limits=c(0,30))+

theme_bw()

# different way to sort

#NCP Phase 2

NCP.2.1<-NCP.2[order(NCP.2$percentage),]

NCP.2.1$'Site' <-factor(NCP.2.1$'Site', levels=NCP.2.1$"Site")

#then plot

ggplot(NCP.2, aes(Site, percentage))+ geom_bar(stat="identity", fill="#00BFC4")+

labs(x="Site", y="Prevalence of Dental Caries (%)") +

scale_y_continuous(limits=c(0,30))+

theme_bw()

#QJ Phase 1

QJ.1.1<-QJ.1[order(QJ.1$percentage),]

QJ.1.1$'Site' <-factor(QJ.1.1$'Site', levels=QJ.1.1$"Site")

ggplot(QJ.1, aes(Site, percentage))+ geom_bar(stat="identity", fill="#F8766D")+

labs(x="Site", y="Prevalence of Dental Caries (%)") +

scale_y_continuous(limits=c(0,30))+

theme_bw()

#QJ Phase 2

QJ.2.1<-QJ.2[order(QJ.2$percentage),]

QJ.2.1$'Site' <-factor(QJ.2.1$'Site', levels=QJ.2.1$"Site")

ggplot(QJ.2, aes(Site, percentage))+ geom_bar(stat="identity", fill="#00BFC4")+

labs(x="Site", y="Prevalence of Dental Caries (%)") +

scale_y_continuous(limits=c(0,30))+

theme_bw()+theme(text=element_text(size=20))+

scale_x_discrete("Site", labels=c("Podi","SG","YQ","Xitun","Qucun","ZTH","DY","SM","ZY","QAC","NZP","SLY","DLS","QJWLJ"))

#GQ both phases

ggplot(GQ, aes(x=Site, y=percentage, fill=as.factor(Phase))) +geom_bar(stat="identity")+

labs(x="Site", y="Prevalence of Dental Caries (%)") +

scale_y_continuous(limits=c(0,30))+

theme_bw() + scale_fill_discrete(name="Phase", labels=c(">4000 BP", "<4000 BP"))

#calculating difference between sexes

library(dplyr)

sum1 <- NCP_S %>%

group_by(Site) %>%

summarize (difference =first(sex_percentage)-last(sex_percentage))

sum2 <- QJ_S %>%

group_by(Site) %>%

summarize (difference =first(sex_percentage)-last(sex_percentage))

sum3 <- GQ_S %>%

group_by(Site) %>%

summarize (difference =first(sex_percentage)-last(sex_percentage))

## plotting sex difference

#attach new datafram

attach(sex_diff)

#sort according to values

sexdiff <- sex_diff[order(sex_diff$diff),]

#convert to factor to retain sorted order in plot

sexdiff$'sitename' <-factor(sexdiff$'sitename', levels= sexdiff$'sitename')

ggplot(sexdiff, aes(x=sitename, y=diff, lab=sitename))+

geom_bar(stat="identity", aes(fill=as.factor(Region)))+

scale_fill_discrete(name="Region",labels=c ("Eastern Central Plains", "Western Central Plains", "Ganqing"))+

coord_flip() + labs(x="Site", y="Difference in Prevalence of Dental Caries between Sexes (%)")

#calculating difference between age groups (only comparing between subadults and older adults)

sum4 <- NCP_A %>%

group_by(Site) %>%

summarize (difference =last(age_percentage)-first(age_percentage))

sum5 <- QJ_A %>%

group_by(Site) %>%

summarize (difference =last(age_percentage)-first(age_percentage))

sum6 <- GQ_A %>%

group_by(Site) %>%

summarize (difference =last(age_percentage)-first(age_percentage))
