# Supplementary material for: Stable isotope and dental caries data reveal abrupt changes in subsistence economy in ancient China in response to global climate change
Source: PLoS One. 2019 Jul 22;14(7):e0218943. doi: 10.1371/journal.pone.0218943 (PMC6645670; doi:10.1371/journal.pone.0218943)
Supplement: S2 File — Principles and caveats of analytical techniques. (DOCX) [file pone.0218943.s002.docx]

S2 - Supplementary Information

Principles and caveats of analytical techniques

1. **Stable isotope analysis**

The concept of stable isotope analysis derives from the fact that the fractionation of each stable isotope varies greatly in the natural world. Generally speaking, the stable isotope patterns of different elements register certain characteristics of the local environment, and through different chemical reactions such as respiration and digestion, these patterns are incorporated into the bodies of different organisms. As the isotopic makeup of an organism should be derived from the corresponding isotopic ratios of its diet, it should be possible to relate an organism to its living environment and diet through stable isotope analysis. Stable carbon (*δ*^13^C) and nitrogen (*δ*^15^N) isotope analysis in particular, is a well-established technique in archaeology to reconstruct dietary practices of past populations (1-3). The *δ*^13^C and *δ*^15^N values of human bone and teeth can help us to reconstruct past dietary patterns. When considered within the larger cultural and geographic context, they can also allow us to make inferences about many aspects of past populations, including changes in long-term subsistence patterns (4-7).

In a terrestrial environment, *δ*^13^C values are primarily used to distinguish between the consumption of C_3_ and C_4_ plants (8). In areas where marine foods are present, *δ*^13^C values can also distinguish between marine and C_3_ terrestrial consumption (1, 8). While *δ*^15^N values are largely used to estimate one’s animal protein intake (9), they can also be influenced by climatic conditions (10), agricultural practices (11, 12), as well as numerous physiological factors such as breastfeeding, starvation, and many more (13-15). Figure S1 shows all the *δ*^13^C values and *δ*^15^N values from all sites discussed in this study. Figure S2 is an isotopic heat map that demonstrates the changes in proportion of C_3_/ C_4_ food consumed across northern China over time.

Note that as *δ*^15^N values are more prone to be affected by external factors, extra measures, both in the sampling stage and the analytical stage, are usually required to account for the different variables. Conventional approaches include only taking samples from certain age group (i.e. adults), avoid sampling from pathological specimen, and using a fauna-derived baseline to assess palaeoclimatic conditions. Unfortunately, not all sites analyzed in this study have provided sufficient background data for us to evaluate the changes in *δ*^15^N values through time. As a result, the trends in *δ*^15^N values will not be discussed in this study.

**Figure S1. Bone collagen *δ*^13^C and *δ*^15^N values of human from all sites discussed in this study: a). *δ*^13^C values of all sites in NCP; b) *δ*^13^C values of all sites in QJ; c) *δ*^13^C values of all sites in GQ; d) *δ*^15^N values of all sites in NCP; e) *δ*^15^N values of all sites in QJ; and f) *δ*^15^N values of all sites in GQ.**


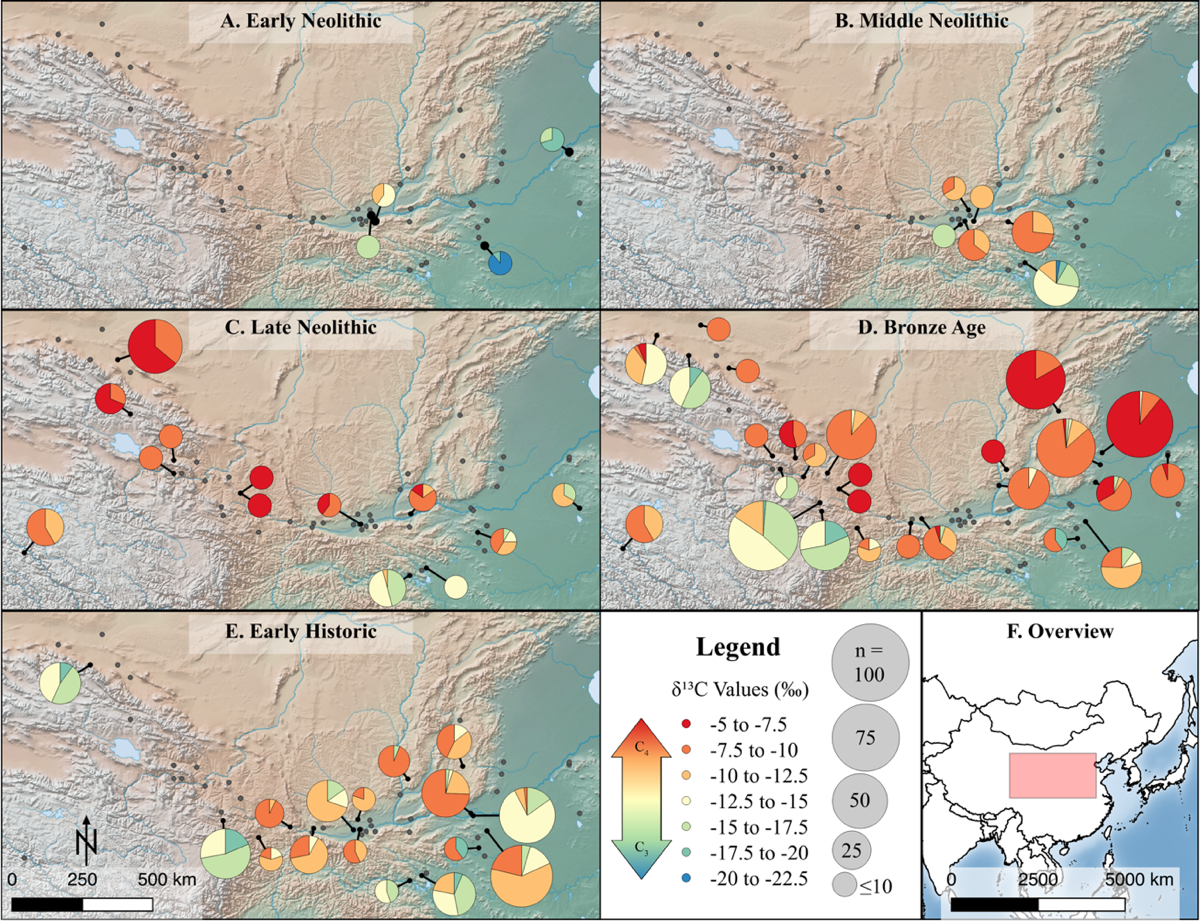


Figure S2. Map demonstrating spatial and temporal trends in *δ*^13^C values. Note that some precision has been lost by binning isotope values. To identity individual sites, use Fig. 1 and Table 2 in main text. Each inset represents a different phase: A) Early Neolithic (9000-7500 BP); B) Middle Neolithic (7500-6000BP); C) Late Neolithic (6000-4500BP); D) Bronze Age (4500-3000BP); E) Early Historical (3000-1750BP); F) Overview of the study area. All maps used are in the public domain (CC0), sourced from Natural Earth ([www.naturalearthdata.com](http://www.naturalearthdata.com)).

1. Dental caries analysis

Dental caries are essentially acid etched lesions on teeth caused by bacterial activity (16). This activity is prompted primarily by the fermentation of dietary sugars (including carbohydrates) in the oral cavity, but the rate can be exacerbated by a number of other factors, such as consumption of soft and sticky food, low saliva pH value and clearance rate (16-20). Accordingly, archaeologists have long associated dental caries and other forms of dental and alveolar defects with the onset of agricultural revolution, where carbohydrates became the major source of caloric intake under this new subsistence economy (21-24). Table S1 shows a detailed summary of dental caries data among different sex and age categories. Figure S3 shows the prevalence of dental caries recorded in all sites discussed in this study.

**Table S1. Summary of caries prevalence in samples of different sex and age categories by tooth count (teeth affected / total of teeth preserved for examination). Populations are further divided into three age groups, after a commonly cited aging method by Mao and Yan ^(41)^ among majority of these studies. NCP refers to North China Plain, QJ refers to the Qinjin region, GQ refers to the Ganqing region.**

| **Phase** | **Year (BP)** | **NCP Sites** | **No. of individuals observed** | **Male** | **Female** | **Subadult**  **(≤20 years)** | **Young adult**  **(20 – 40 years)** | **Older adult**  **(≥40 years)** |
| --- | --- | --- | --- | --- | --- | --- | --- | --- |
| >4000BP | 7000-6000 | Xiawanggang | 161 | 40 / 710 | 19 / 265 | 1 / 50 | 19 / 662 | 32 / 263 |
| >4000BP | 6000-5000 | Gouwan | 56 | 25 / 416 | 29 / 285 | － | － | － |
| >4000BP | 6500-4500 | Qinglongquan | 87 | － | － | － | － | － |
| >4000BP | 6100-4500 | Dawenkou | 17 | － | － | － | － | － |
| >4000BP | 5600-5000 | Guangwu | 15 | － | － | － | － | － |
| <4000BP | 3400-3100 | Yinxu | 118 | 49 / 1355 | 33 / 642 | 1 / 306 | 39 / 835 | 23 / 441 |
| <4000BP | 3000-2220 | Chenjiagou | 61 | 69 / 227 | 76 / 353 | 0 / 42 | 13 / 198 | 123 / 376 |
| <4000BP | 2000-1800 | Panmiao | 17 | 19 / 156 | 6 / 82 | － | － | － |
|  |  | **QJ Sites** |  |  |  |  |  |  |
| >4000BP | 7000-6000 | Baoji Huaxian (BJHX) | 59 | 23 / 775 | 9/ 173 | 1 / 208 | 26 / 447 | 5 / 187 |
| >4000BP | 7000-6000 | Jiangzhai  (JZ) | 37 | 5 / 255 | 6 / 163 | － | － | － |
| >4000BP | 7000-5790 | Beishouling (BSL) | 36 | － | － | － | － | － |
| >4000BP | 6390 | Beiliu | 10 | 3 / 46 | 1 / 46 | － | － | － |
| >4000BP | 6500-5500 | Banpo  (BP) | 73 | － | － | 4 / 151 | 7 / 389 | 13 / 359 |
| >4000BP | 6000-5000 | Shijia | 49 | 5 / 134 | 2 / 45 | － | － | － |
| >4000BP | 5500-5000 | Duzhong (DZ) | 15 | 33 / 297 | 10 / 79 | 21 / 174 | 9 / 90 | 15 / 112 |
| >4000BP | 4900-4800 | Miaodigou  (MDG) | 10 | － | － | － | － | － |
| >4000BP | 4500-4000 | Kangjia | 16 | 27 / 92 | 15 / 54 | － | － | － |
| >4000BP | 4500-3900 | Taosi | 180 | 11 / 1888 | 17 / 1007 | 0 / 203 | 7 / 1413 | 21 / 1279 |
| <4000BP | 4000-3600 | Sanguan  (SG) | 7 | － | － | － | － | － |
| <4000BP | 3900-3500 | Donglongshan  (DLS) | 24 | 15 / 123 | 20 / 121 | 0 / 5 | 22 / 119 | 13 / 120 |
| <4000BP | 3100-2800 | Nianzipo  (NZP) | 111 | 124 / 1231 | 75 / 508 | 2 / 234 | 100 / 1080 | 97 / 425 |
| <4000BP | 3100-2800 | Shaolingyuan  (SLY) | 147 | 82 / 737 | 133 / 946 | 9 / 326 | 74 / 556 | 94 / 638 |
| <4000BP | 3100-2800 | Zhouyuan  (ZY) | 25 | 38 / 326 | 25 / 341 | 1 / 90 | 33 / 416 | 29 / 161 |
| <4000BP | 3100-2800 | Qucun | 25 | 23 / 351 | 10 / 132 | 4 / 90 | 23 / 234 | 17 / 159 |
| <4000BP | 3100-2000 | Dongyang  (DY) | 47 | 24 / 276 | 31 / 450 | 1 / 142 | 20 / 384 | 34 / 200 |
| <4000BP | 2800-2500 | Yanqing  (YQ) | 171 | 182 / 2048 | 159 / 1719 | 0 / 305 | 216 / 2656 | 125 / 806 |
| <4000BP | 2800-2500 | Shangma  (SM) | 130 | 78 / 1244 | 88 / 1304 | 1 / 265 | 67 / 1355 | 98 / 928 |
| <4000BP | 2500-2200 | Zhaitouhe  (ZTH) | 23 | － | － | － | － | － |
| <4000BP | 2500-2200 | Qujia Wanlijia  (QJWLJ) | 83 | 135 / 590 | 79 / 412 | 8 / 142 | 81 / 410 | 99 / 385 |
| <4000BP | 2500-2000 | Qiaocun  (QAC | 514 | 423 / 3867 | 370 / 3087 | 17 / 884 | 401 / 3969 | 375 / 2101 |
| <4000BP | 2200-2000 | Podi | 111 | 13 / 428 | 17 / 647 | 3 / 509 | 33 / 711 | 14 / 297 |
| <4000BP | 2000-1800 | Xitun | 201 | － | － | － | － | － |
|  |  | **GQ Sites** |  |  |  |  |  |  |
| >4000BP | 4400-4000 | Liuwan | 53 | － | － | － | － | － |
| <4000BP | 4400 | Mogou | 223 | 86 / 1898 | 208 / 2372 | 19 / 1184 | 149 / 2792 | 130 / 917 |
| <4000BP | 3000-2200 | Xishan | 22 | － | － | － | － | － |

Figure S3. Prevalence of dental caries organized by period and site, note that the sites are not organized in chronological order in each phase, for abbreviations of site names please refer to Table S1.

Potential error in dental caries analysis

Herein we provide a brief overview of several relevant sources of potential error in dental caries analysis, and how they could affect our analysis. Many of these issues are caused by the lack of standardization in data reporting, as well as often unclear descriptions of methodological approaches. While the quality of our analysis is ultimately depending on the quality of the data we used, the “look-back” nature of our study meant that there is very little we can do to improve the original data. However, this particular problem should not stop us from analyzing these data, but we should proceed with utmost caution with these issues in mind.

*Inter-observer bias*

Archaeologists are acutely aware of how the subjectivity of a recorder can affect the integrity of qualitative research. This is especially true in analyzing dental caries data, where the severity of caries is often misclassified, and in some cases, the presence or absence of carious lesion completely misidentified (25, 26). Unfortunately, all of the data collected and discussed in this paper are obtained from published reports, many of which did not clearly state their methodological approach, and how they address, and correct for the potential inconsistency caused by inter-observer bias. Thus, all collated data are reported on an as-is basis, under the assumption that all reports and peer-reviewed articles meet academic standards for practices and data quality.

*Morphological and pathological factors*

One common problem in assessing the prevalence of dental caries among archaeological populations is the need to account for other morphological as well as pathological conditions that could affect the occurrence of caries.

Molars are more susceptible to carious lesions than other teeth due to its shape and functions (27, 28). However, their larger sizes and often curved roots meant that molars tend to be better preserved than other types of teeth (29). As a result, when accounting for prevalence of dental caries in a population using overall tooth count, we risk inflating the actual rate of dental caries within a population. Again, due to the different styles of data reporting, we are not able to account for how the variations in tooth representation could potentially affect the calculation of prevalence of carious lesion.

Other dental pathological conditions, such as dental calculus (plague), dental attritions, and periodontal diseases, are not necessarily causative of dental caries, but the pattern and severity of these conditions can often offer valuable insights into the cause of carious lesions, for example whether it was dietary, or lifestyle related (28, 30-32). The lack of standardization in dental data reporting in archaeology meant that not all studies report detailed dental pathological data, making it very difficult to fully understand the prevalence of dental caries in any particular population. While the lack of detailed dental pathological data is definitely a setback in building a holistic picture of how different dietary practices could affect dental health, plenty of odontogological studies have demonstrated that with a substantial sample size and a conservative assessment approach, dental health data, and in particular, dental caries data, can still provide valuable insights into the subsistence strategies of past populations.

Additionally, it is important to understand that ante-mortem tooth loss (AMTL) can be caused by severe carious decay. Several studies have attempted to solve this issue by proposing various mathematical models to correction for the error(33-35). This approach has proven to be successful in many case studies, and has significantly improved the correlation in prevalence of dental caries between males and females (36, 37). Unfortunately, not all of reports discussed in our paper provide detailed information on individual odontological conditions, therefore, it is not possible to apply the correction factor on all sites. Thus, instead of only running the correction model on some of the sites, we have decided to not correct for AMTL at all to keep our data comparable.

*Sex and Age*

Numerous studies have already identified that due to a number of genetic, physiological, and behavioural factors, females (38-40), as well as older individuals (28, 41, 42), are more prone to dental caries than males and younger individuals. In this study, a similar trend is observed, where two-third (16/24) of the sites with sexing information reported a higher prevalence of dental caries among females than males (Table S1, Figure S4, and Figure S5), and that the prevalence of dental caries in older adults are significantly higher than those in subadults across all sites with age information (Table S3 and Figure S6).

Table S2. Difference in the prevalence of dental caries between sexes at all sites. Positive values indicate higher prevalence among males, negative values indicate higher prevalence among females, – indicate no sexing data available.

|  | **NCP** |  | **QJ** |  | **GQ** |  |
| --- | --- | --- | --- | --- | --- | --- |
|  | **Site** | **Diff (%)** | **Site** | **Diff (%)** | **Site** | **Diff (%)** |
|  | Chenjiagou | +8.9 | Banpo (BP) | – | Liuwan | – |
|  | Dawenkou | – | Baoji Huaxian (BJHX) | –2.2 | Mogou | –4.2 |
|  | Gouwan | –4.2 | Beiliu (BL) | +4.3 | Xishan | – |
|  | Guangwu | – | Beishouling (BSL) | – |  |  |
|  | Panmiao | +4.8 | Donglongshan (DLS) | –4.3 |  |  |
|  | Qinglongquan | – | Dongyang (DY) | +1.8 |  |  |
|  | Xiawanggang | –1.5 | Duzhong (DZ) | –1.5 |  |  |
|  | Yinxu | –1.5 | Jiangzhai (JZ) | –1.7 |  |  |
|  |  |  | Kangjia (KJ) | +1.6 |  |  |
|  |  |  | Miaodigou (MDG) | – |  |  |
|  |  |  | Nianzipo (NZP) | –4.7 |  |  |
|  |  |  | Podi (PD) | +0.4 |  |  |
|  |  |  | Qi Aocun (QAC) | –1.0 |  |  |
|  |  |  | Qucun (QC) | –1.0 |  |  |
|  |  |  | Qujia Wanlijia (QJWLJ) | +3.7 |  |  |
|  |  |  | Sanguan (SG) | – |  |  |
|  |  |  | Shangma (SM) | –0.4 |  |  |
|  |  |  | Shaolingyuan (SLY) | –2.5 |  |  |
|  |  |  | Shijia (SJ) | –0.7 |  |  |
|  |  |  | Taosi (TS) | –1.1 |  |  |
|  |  |  | Xitun (XT) | – |  |  |
|  |  |  | Yanqing (YQ) | –0.5 |  |  |
|  |  |  | Zhaitouhe (ZTH) | – |  |  |
|  |  |  | Zhouyuan (ZY) | +4.3 |  |  |
|  |  |  |  |  |  |  |
| **Average** |  | **+1.3** |  | **–0.3** |  | **–4.2** |

Figure S4. Prevalence of dental caries in the three regions by sex organized by scale of difference in prevalence (%), positive values indicate higher prevalence among males, negative values indicate higher prevalence among females.

Figure S5. Prevalence of dental caries in the three regions by sex, for abbreviations of site names please refer to Table S2.

Table S3. Difference in the prevalence of dental caries between subadults and older adults at all sites, positive values indicate higher prevalence among older adults, negative values indicate higher prevalence among subadults.

|  | **NCP** |  | **QJ** |  | **GQ** |  |
| --- | --- | --- | --- | --- | --- | --- |
|  | Site | Diff (%) | Site | Diff (%) | Site | Diff (%) |
|  | Chenjiagou | +32.7 | Banpo (BP) | +1.0 | Liuwan | – |
|  | Dawenkou | – | Baoji Huaxian (BJHX) | +2.2 | Mogou | +12.6 |
|  | Gouwan | – | Beiliu (BL) | – | Xishan | – |
|  | Guangwu | – | Beishouling (BSL) | – |  |  |
|  | Panmiao | – | Donglongshan (DLS) | –4.3 |  |  |
|  | Qinglongquan | – | Dongyang (DY) | +1.8 |  |  |
|  | Xiawanggang | +10.2 | Duzhong (DZ) | +1.3 |  |  |
|  | Yinxu | +4.9 | Jiangzhai (JZ) | –1.7 |  |  |
|  |  |  | Kangjia (KJ) | +1.6 |  |  |
|  |  |  | Miaodigou (MDG) | – |  |  |
|  |  |  | Nianzipo (NZP) | +22.0 |  |  |
|  |  |  | Podi (PD) | +4.1 |  |  |
|  |  |  | Qi Aocun (QAC) | +15.9 |  |  |
|  |  |  | Qucun (QC) | +6.2 |  |  |
|  |  |  | Qujia Wanlijia (QJWLJ) | +20.1 |  |  |
|  |  |  | Sanguan (SG) | – |  |  |
|  |  |  | Shangma (SM) | +15.5 |  |  |
|  |  |  | Shaolingyuan (SLY) | +11.9 |  |  |
|  |  |  | Shijia (SJ) | – |  |  |
|  |  |  | Taosi (TS) | +1.6 |  |  |
|  |  |  | Xitun (XT) | – |  |  |
|  |  |  | Yanqing (YQ) | +10.2 |  |  |
|  |  |  | Zhaitouhe (ZTH) | – |  |  |
|  |  |  | Zhouyuan (ZY) | +16.9 |  |  |
|  |  |  |  |  |  |  |
| **Average** |  | **+15.9** |  | **+9.6** |  | **+12.6** |

Figure S6. Prevalence of dental caries in the three regions by age, for abbreviations of site names please refer to Table S3.

As shown in Table S2 and Table S3, the magnitude of difference varies greatly from site to site, possibly due to the diversity of diets and socio-cultural behaviours practiced by these different populations. While some scholars advocate only comparing prevalence of dental caries between corresponding age and sex groups (28, 43), we feel that for the purpose of this study, a general average from each site is sufficiently representative for the estimation of general tendency of dental caries prevalence at each site. In fact, among sites with sexing and/or age information, young adult males dominate the demographic makeup (18/19 sites have a higher number of subadults and young adults; 15/24 sites have a higher number of males). Thus, most of the sites compared in this study have roughly similar demographic compositions.

1. **Sites with overlapping stable isotopic and dental data**

There are only 11 sites with overlapping stable isotopic and dental caries data. A list of all these sites, summary of *δ*^13^C values as well as dental caries data are presented in Table S4. Unfortunately, these data are too temporally and spatially scattered to be evaluated in a meaningful way.

Table S4. Sites with overlapping stable isotopic and dental caries data. Note that the stable isotopic data from the last phase of Qinglongquan (n=9) and Gouwan (n=1) were excluded, this is to ensure all data are generated from comparable populations.

| **Site** | **Region** | **Period (BP)** | **Prevalence of dental caries (%)** | ***δ*^13^C (‰, VPDB)** |
| --- | --- | --- | --- | --- |
| Mogou | GQ | 4000-3500 | 6.1% (n=223) | –14.4±1.73 (n=84) |
| Xishan | GQ | 3500-1800 | 10.7% (n=22) | –11.4±2.08 (n=19) |
|  |  |  |  |  |
| Beiliu | QJ | 8500-5500 | 4.4% (n=10) | –11.8±1.25 (n=9) |
| Banpo | QJ | 7000-6500 | 2.9% (n=73) | –15 (n=1) |
| Jiangzhai | QJ | 7000-6000 | 2.6% (n=37) | –9.7±0.97 (n=17) |
| Shijia | QJ | 6500-5000 | 3.9% (n=49) | –10.0±0.68 (n=9) |
| Zhouyuan | QJ | 3500-3000 | 9.3% (n=25) | –9.9±1.91 (n=20) |
|  |  |  |  |  |
| Gouwan | NCP | 7000-5500 | 7.2% (n=56) | –14.4±2.00 (n=36) |
| Qinglongquan | NCP | 6500-4000 | 11.9% (n=87) | –14.6±1.25 (n=24) |
| Yinxu | NCP | 3500-3000 | 4.1% (n=118) | –9.1±1.35 (n=59) |
| Chenjiagou | NCP | 3000-2500 | 22.9% (n=61) | –9.7±1.47 (n=39) |

### References

1. Katzenberg M. Stable Isotope Analysis: A Tool of Studying Past Diet, Demography, and Life History. In: Katzenberg M, Saunders S, editors. Biological Anthropology of the Human Skeleton. Second Edition ed. Hoboken, NJ: John Wiley & Sons, Inc.; 2008. p. 413-41.

2. Schoeninger M, Moore K. Bone Stable Isotope Studies in Archaeology. Journal of World Prehistory. 1992;6(2):246-96.

3. Schwarcz H, Schoeninger M. Stable Isotopes of Carbon and Nitrogen as Tracers for Paleo-Diet Reconstruction. In: Baskaran M, editor. Handbook of Environmental Isotope Geochemistry. Advances in Isotoep Geochemistry. 1. Heidelberg: Springer; 2011. p. 725-42.

4. Liu X, Lightfoot E, O'Connell T, Wang H, Li S, Zhou L, et al. From Necessity to Choice: Dietary Revolutions in West China in the Second Millennium BC. World Archaeology. 2014;46(5):661-80.

5. Katzenberg M, Schwarcz H, Knyf M, Melbye F. Stable Isotope Evidence for Maize Horticulture and Palaeodiet in Southern Ontario, Canada. American Antiquity. 1995;60(2):335-50.

6. Zhou L, Garvie-Lok S. Isotopic Evidence for the Expansion of Wheat Consumption in Northern China. Archaeological Research in Asia. 2015:25-35.

7. Miller A, Usmanova E, Logvin V, Kalieva S, Shevnina I, Logvin A, et al. Subsistence and Social Change in Central Eurasia: Stable Isotope Analysis of Populations Spanning the Bronze Age Transition. Journal of Archaeological Science. 2014;42:525-38.

8. Boutton T. Stable Carbon Isotope Ratios of Natural Materials: II. Atmospheric, Terrestrial, Marine, and Freshwater Environments. In: Coleman D, editor. Carbon Isotope Techniques. San Diego: Academic Press; 1991. p. 173-86.

9. Hedges R, Reynard L. Nitrogen Isotopes and the Trophic Level of Humans in Archaeology. Journal of Archaeological Science. 2007;34(8):1240-51.

10. Gröcke D, Bocherens H, Mariotti A. Annual Rainfall and Nitrogen-Isotope Correlation in Macropod Collagen: Application as a Palaeoprecipitation Indicator. Earth and Planetary Science Letters. 1997;153(3):279-85.

11. Bogaard A, Heaton T, Poulton P, Merbach I. The Impact of Manuring on Nitrogen Isotope Ratios in Cereals: Archaeological Implications for Reconstruction of Diet and Crop Management Practices. Journal of Archaeological Science. 2007;34(3):335-43.

12. Szpak P, Millaire J-F, White C, Longstaffe F. Influence of Seabird Guano and Camelid Dung Fertilization on the Nitrogen Isotopic Composition of Field-Grown Maize (Zea Mays). Journal of Archaeological Science. 2012;39(12):3721-40.

13. Fuller B, Molleson T, Harris D, Gilmour L, Hedges R. Isotopic Evidence for Breastfeeding and Possible Adult Dietary Differences from Late/Sub-Roman Britain. American Journal of Physical Anthropology. 2006;129:45-54.

14. Reitsema L. Beyond Diet Reconstruction: Stable Isotope Applications to Human Physiology, Health, and Nutrition. American Journal of Human Biology. 2013;25(4):445-56.

15. Hobson K, Alisauskas R, Clark R. Stable Nitrogen Isotope Enrichment in Avian Tissue Due to Fasting and Nutritional Stress: Implications for Isotopic Analysis of Diet. The Condor. 1993;95(2):388-94.

16. Selwitz R, Ismail A, Pitts N. Dental Caries. The Lancet. 2007;369(9555):51-9.

17. Moynihan P. Sugars and Dental Caries: Evidence for Setting a Recommended Threshold for Intake. Advances in Nutrition. 2016;7(1):149-56.

18. Gupta P, Gupta N, Pawar A, Birajdar S, Natt A, Singh H. Role of Sugar and Sugar Substitutes in Dental Caries: A Review. ISRN Dentistry. 2013;2013:5.

19. Lingstrom P, van Houte J, Kashket S. Food Starches and Dental Caries. Critical Reviews in Oral Biology & Medicine. 2000;11(3):366-80.

20. Tayles N, Domett K, Halcrow S. Can Dental Caries be Interpreted as Evidence of farming? The Asian Experience. In: Koppe T, Meyer G, Alt KW, editors. Comparative Dental Morphology. 13: Karger Publishers; 2009. p. 162-6.

21. Larsen C. Biological Changes in Human Populations with Agriculture. Annual Review of Anthropology. 1995;24:185-213.

22. Cohen M, Armelagos G. Paleopathology at the Origins of Agriculture. Orlando: Academic Press New York; 1984 1984. 615 p.

23. Powell M. The Analysis of Dental Wear and Caries for Dietary Reconstruction. In: Gilbert R, Mielke J, editors. Analysis of Prehistoric Diets. Orlando: Academic Pres; 1985. p. 307-38.

24. Temple D, Larsen C. Dental Caries Prevalence as Evidence for Agriculture and Subsistence Variation during the Yayoi Period in Prehistoric Japan: Biocultural Interpretations of an Economy in Transition. American Journal of Physical Anthropology. 2007;134(4):501-12.

25. Lesaffre E, Mwalili S, Declerck D. Analysis of Caries Experience Taking Inter-observer Bias and Variability into Account. Journal of Dental Research. 2004;83(12):951-5.

26. Liebe-Harkort C, Ástvaldsdóttir Á, Tranaeus S. Quantification of dental caries by osteologists and odontologists-a validity and reliability study. International Journal of Osteoarchaeology. 2009;20(5):n/a-n/a.

27. Demirci M, Tuncer S, Yuceokur A. Prevalence of Caries on Individual Tooth Surfaces and its Distribution by Age and Gender in University Clinic Patients. European Journal of Dentistry. 2010;4(3):270-9.

28. Hillson S. Recording Dental Caries in Archaeological Human Remains. International Journal of Osteoarchaeology. 2001;11(4):249-89.

29. Jackes M. Representativeness and Biasness in Archaeological Skeletal Samples. In: Agarwal S, Glencross B, editors. Social Bioarchaeology. Chichester: Blackwell Publishing; 2011. p. 107-46.

30. Axelsson P, Nyström B, Lindhe J. The Long-Term Effect of a Plaque Control Program on Tooth Mortality, Caries and Periodontal Disease in Adults. Journal of Clinical Periodontology. 2004;31(9):749-57.

31. Scannapieco F, Torres G, Levine M. Salivary α-Amylase: Role in Dental Plaque and Caries Formation. Critical Reviews in Oral Biology & Medicine. 1993;4(3):301-7.

32. Maat G, Van der Velde E. The Caries-Attrition Competition. International Journal of Anthropology. 1987;2(4):281.

33. Lukacs JR. The ‘caries correction factor’: A new method of calibrating dental caries rates to compensate for antemortem loss of teeth. International Journal of Osteoarchaeology. 1995;5(2):151-6.

34. Duyar I, Erdal YS. A new approach for calibrating dental caries frequency of skeletal remains. Homo. 2003;54(1):57-70.

35. Erdal Y, Duyar İ. A New Correction Procedure for Calibrating Dental Caries Frequency. American Journal of Physical Anthropology. 1999;108(2):237-40.

36. Tayles N, Domett K, Nelsen K. Agriculture and Dental Caries? The Case of Rice in Prehistoric Southeast Asia. World Archaeology. 2000;32(1):68-83.

37. Domett K. Health in late prehistoric Thailand. Oxford: Archaeopress; 2001 2001.

38. Lukacs J, Largaespada L. Explaining Sex Differences in Dental Caries Prevalence: Saliva, Hormones, and "Life-History" Etiologies. American Journal of Human Biology. 2006;18(4):540-55.

39. Lukacs JR. Gender differences in oral health in South Asia: metadata imply multifactorial biological and cultural causes. Am J Hum Biol. 2011;23(3):398-411.

40. Lukacs JR, Thompson LM. Dental caries prevalence by sex in prehisotory: magnitude and meaning. In: Irish JD, Nelson GC, editors. Technique and Application in Dental Anthropology. Cambridge: Cambridge University Press; 2008. p. 136-77.

41. Gati D, Vieira A. Elderly at Greater Risk for Root Caries: A Look at the Multifactorial Risks with Emphasis on Genetics Susceptibility. International Journal of Dentistry. 2011;2011(Article ID 647168):6 pages.

42. Limbo J. The Frequency and Pattern of Dental Caries in Archaeological Populations from Estonia. Papers on Anthopology. 2013;XXII:121-32.

43. Sakashita R, Inoue M, Inoue N, Pan Q, Zhu H. Dental Diseases in the Chinese Yin-Shang Period with Respect to Relationships between Citizens and Slaves. American Journal of Physical Anthropology. 1997;103(3):301-408.
